# Therapeutic effect of small extracellular vesicles from cytokine-induced memory-like natural killer cells on solid tumors

**DOI:** 10.1186/s12951-024-02676-1

**Published:** 2024-07-29

**Authors:** Yinghong Shi, Yanxia Chen, Yi Wang, Dan Mo, Huisheng Ai, Jianguo Zhang, Mei Guo, Hui Qian

**Affiliations:** 1 Jiangsu Province Key Laboratory of Medical Science and Laboratory Medicine, Department of Laboratory Medicine, School of Medicine, Jiangsu University, Zhenjiang, 212013 China; 2 Zhenjiang Municipal Key Laboratory of High Technology for Basic and Translational Research on Exosomes, Zhenjiang, 212013 China; 3 Department of Hematology, The Fifth Medical Center, Chinese PLA General Hospital, Beijing, 100071 China; 4 Department of Emergency Medicine, The Affiliated Hospital, Jiangsu University, Zhenjiang, 212001 Jiangsu China

**Keywords:** Small extracellular vesicle, Memory-like natural killer cells, Granulysin, Cytotoxicity, Cancer therapy

## Abstract

**Abstract:**

Small extracellular vesicles (sEV) derived from diverse natural killer (NK) cell lines have proven their exceptional antitumor activities. However, sEV from human primary NK cells, especially memory-like NK cells, are rarely utilized for cancer treatment. In this study, we obtained sEV from IL-12, IL-15 and IL-18 cultured human memory-like NK cells (mNK-sEV) that showed strong cytokine-secretory ability. It was uncovered that mNK-sEV entered cancer cells via macropinocytosis and induced cell apoptosis via caspase-dependent pathway. Compared to sEV from conventionally cultured NK cells (conNK-sEV), mNK-sEV inhibited tumor growth to a greater extent. Concomitantly, pharmacokinetics and biodistribution results validated a higher accumulation of mNK-sEV than conNK-sEV in tumors of xenografted murine models. Notably, elevated containment of granulysin (GNLY) within mNK-sEV, at least in part, may contribute to the enhanced therapeutic effect. Herein our results present that mNK-sEV can be a novel class of therapeutic reagent for effective cancer treatment.

**Graphical Abstract:**

**Figure:**
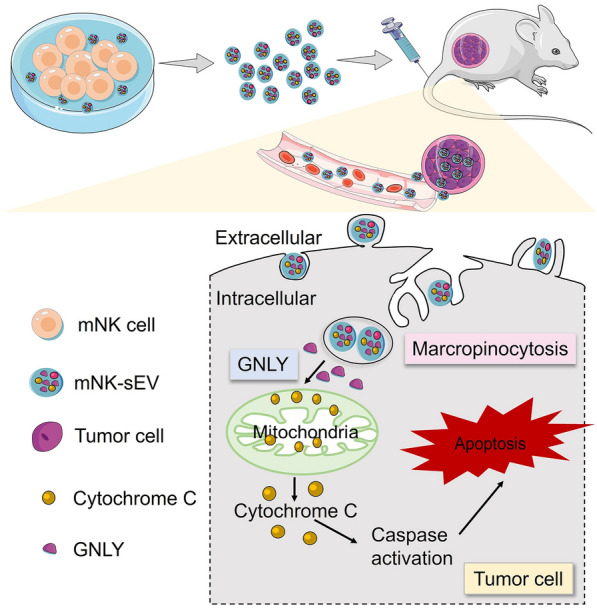

**Supplementary Information:**

The online version contains supplementary material available at 10.1186/s12951-024-02676-1.

## Background

Natural killer (NK) cells are innate cytotoxic effectors, marked by absence of T cell receptor and associated CD3 molecules and by expression of neural cell adhesion molecule known as CD56 [1]. They possess strong cytolytic function and can kill tumor cells without antigen presentation [2]. The effector function of NK cells is governed by the activating and inhibiting receptors that can distinguish tumor cells from healthy cells [3]. Simultaneously, NK cells secrete cytotoxic granules (e.g., perforin, granzymes), cytokines (e.g., IFN-γ, TNF-α, TNF-β, IL-6, GM-CSF) and chemokines (e.g., CCL3, CCL4, CCL5), which may further activate T cells and B cells and influence the function of macrophages, dendritic cells, and neutrophils, finally destructing tumors [4, 5]. For these reasons, NK cells have been actively applied in the cell-based cancer treatment [6]. However, clinical trials of NK cell therapy have reported mixed results, where limited tumor infiltration and immunosuppressive phenotype lowered the antitumor potency of NK cells, especially in solid tumors [7, 8].

Of diverse biogenic origins, small extracellular vesicles (sEV) with diameters of 30–200 nm are a subgroup of extracellular vesicles (EV) traditionally referred as exosomes [9]. They are double-layer membrane vesicles released from nearly all cell types, containing a variety of bioactive components [10]. Compared to engineered nanomaterials, sEV have shown many superior physicochemical and biological aspects, such as unmatched biocompatibility, enhanced capacity to cross biological barriers, minimized recognition by reticuloendothelial system and specific tissue targeting [11]. For instance, compared to liposomes of similar size, CD47^high^ sEV derived from human foreskin fibroblasts and loaded with therapeutic siRNA showed much longer circulation and higher retention in mice bearing KRAS^G12D^ orthotopic pancreatic cancers, significantly increasing metastatic suppression and survival rate [12]. Cytotoxic CD8^+^ T cell-derived EV were found to effectively penetrate into tumor stroma and induce tumor cell apoptosis, so attenuating tumor progression and metastasis [13]. For these reasons, sEV have been actively applied as therapeutical biologics against cancers in preclinical and clinical settings [14, 15].

Studies demonstrated that sEV derived from NK cells owned substantial capacities including tumor accumulation, immune activation, and anti-tumor activity [16, 17]. With efficacy NK cell-derived sEV treated a range of xenografted malignant tumors including glioblastoma and melanoma, which generally inherited from their parental cells [18, 19]. Those tumor-killing effects may pave them a new avenue in the domain of cancer immunotherapy. Nevertheless, given the heterogeneity of NK cells, it is intriguing to explore more therapeutic modalities of their derived sEV against cancers. For example, sEV from NK92 cell lines, NK3.3 cell lines or primary NK cells under various conditions differed in their biological components, so exhibiting distinctive antitumor effects [20]. Cytokine-induced memory-like (CIML) NK cells (mNK) were produced from a feeder-free NK cell expansion system with cytokines IL-12, IL-15, and IL-18 primed [21]. Compared to non-preactivated NK cells, CIML NK cells exhibited higher IFN-γ production upon cytokine restimulation [22]. Besides an enhanced reactivity, there are many alterations in their phenotype. Remarkably, CIML NK cells constitute a more activated phenotype than control NK cells, such as the elevated expression of CD25 and CD69 [23]. In addition, phenotypic characterization of activating receptors and inhibiting receptors on CIML NK cells revealed enhanced expression of NKG2D, NKp46, NKp44 and NKp30 and decreased expression of KIR2D [24]. Specifically, CIML NK cells undergone metabolic reprogramming by upregulating the expression of the heavy subunit of multiple heterodimeric amino acid transporters CD98, and glucose transporters GLUT1 [25]. They displayed enhanced therapeutic effects against myeloid leukemia than conventionally cultured NK cells (conNK) [26, 27].

Despite these developments, the biological characters and potential functions of sEV derived from mNK cells (i.e., mNK-sEV) against tumors, especially solid tumors, are unclear. Moreover, the mechanism regarding how mNK-sEV exerted their anticancer activity remains hidden, and whether their therapeutic efficacy is superior to that of sEV from conNK (i.e., conNK-sEV) waits to be further elucidated. In this study, we isolated sEV from cytokine primed mNK cells and investigated the therapeutic role and mechanism of mNK-sEV in treating cancers both in vitro and in vivo, with comparison to those of conNK-sEV. Through this study we provide significant evidence in support of antitumor activity of mNK-sEV and corroborate their potential as a new immunotherapeutic strategy for clinical cancer treatment.

## Materials and methods

### Mice and cell lines

Pathogen-free 6-week-old female BALB/c nude mice were obtained from Cavens Inc. (Suzhou, China). All animal experimental protocols were approved by the animal care and use committee of Jiangsu University. All animal experiments were performed in accordance with the associated relevant guidelines and regulations for working with live animals. Human gastric cancer cell line MGC803, human non-small-cell lung cancer cell line A549, human pancreatic cancer cell line Patu8988t and human proximal convoluted tubule epithelial cells of renal cortex cell line HK-2 were purchased from the cell bank of the Chinese Academy of Sciences (Shanghai, China). MGC803 cells and Paut8988t cells were cultured in Dulbecco’s modified Eagle’s medium (BI, Israel), and A549 cells and HK-2 cells were cultured in F12 medium (BI, Israel). Chronic myeloid leukemia cell line K562 was a gift from Research Institute for Cancer Therapy, The First Affiliated Hospital, China Medical University, Shenyang, China, and was cultured in RPMI1640 medium (BI, Israel). All cells were cultured with 10% fetal bovine serum (Gibco, USA) and 1% penicillin–streptomycin (Hyclone, USA) at 37 °C in a humidified 5% CO_2_ atmosphere.

### NK cell isolation and culture

Normal peripheral blood mononuclear cells (PBMCs) were obtained from healthy donors in Department of Hematology and Transplantation, The Fifth Medical Center, Chinese PLA General Hospital. Informed consents were obtained from healthy donors, and the study was approved by the Ethics Committee of the Fifth Medical Center, PLA General Hospital, Beijing, China. The research is conducted in accordance with the Declaration of Helsinki. To generate memory-like and control NK cells, PBMCs were first primed with rhIL-12 (10 ng/mL) plus rhIL-18 (50 ng/mL) and rhIL-15 (50 ng/mL), or cultured under control conditions (rhIL-2 10 ng/mL) for 16 ± 2 h, then cultured in complete L15 medium (Lonza, Switzerland) containing 10% human AB serum (Sigma, USA) and 1% penicillin–streptomycin (Hyclone, USA) supplemented with rhIL-2 (1 ng/mL) to support survival. Half of the medium was replaced every 2 days with fresh cell medium supplemented with rhIL-2 [27]. Meanwhile, we calculated total number of NK cells every 2 days to monitor their proliferation. After fourteen days, NK cells were further purified from the culture with CD56 MicroBeads (Miltenyi Biotec, Germany). NK cells (CD3^−^CD56^+^ ≥ 90%) were used for the following experiments. PMBCs extracted from 16 donors were used to culture and isolate NK cells for their derived sEVs.

### Isolation and characterization of NK cells-derived sEV

NK cells were cultured in L15 medium supplemented with 10% of EV-depleted fetal bovine serum (FBS) (Thermo Fisher Scientific, USA) for 48 h. The process to isolate sEV from the collected supernatant was previously reported [28]. In brief, 500 mL supernatant was centrifuged at 1000*g* for 10 min to remove cell debris, then at 10,000*g* for 30 min to remove organelle, followed by 30 min at 2000*g* at 4 ℃ using 100 kDa MWCO before the concentrated solutions were filtrated through a 0.22-µm pore filter (Millipore, USA). sEV was then precipitated by utilizing the exosome quick extraction solution (Millipore, USA), resuspended in 10–30 mL phosphate buffered saline (PBS), stored at − 80 ℃ and used immediately or within 1 week. For characterization, the protein concentration of sEV was determined 5–10 mg/mL by using bicinchoninic acid (BCA) protein assay kit (Vazyme, China). To confirm the successful isolation and purification of sEV, specific biomarkers CD9, CD63, Alix and cytochrome c (1:500) (Cell Signaling Technology (CST), USA) were determined by Western blotting. The morphology, size distribution and zeta potential of sEV were identified using transmission electron microscopy (TEM) (Philips FEI Tecnai 12, Netherlands) and Nanosight tracking analysis (NTA) (NanoSight, UK).

### Cell viability assay

The cytotoxicity of mNK-sEV against cells was evaluated by CCK-8 assay (Vazyme, China). Cells were seeded in 96-well plates overnight at 37 ℃. Various concentrations of mNK-sEV were added into the culture plates for 12, 24 and 48 h. Before the measurement the culture media of different groups were discarded, and fresh medium containing 10% (v/v) CCK-8 solution was added into 96-well plates for 2 h. Absorbance of each well was measured at 450 nm by using an enzyme-linked immunosorbent plate assay reader (FLX800, USA). For comparison, cell viability of tumor cells was evaluated by CCK-8 assay after different treatments including PBS, conNK-sEV (100 µg/mL) and mNK-sEV (100 µg/mL). All EV concentrations applied in this study were presented as the concentrations of total proteins.

### Cell apoptosis assay

To confirm the cytotoxic effects of mNK-sEV on cells, Annexin V-APC/7-AAD double staining was performed with Annexin V-APC/7-AAD Apoptosis Detection Kit (Vazyme, China). Cells were seeded in 96-well plates (5 × 10^4^ cells per well) overnight at 37 ℃. Various concentrations of mNK-sEV (100 and 200 µg/mL) were added into the culture plates for 24 h. After incubation, cells were harvested, stained and analyzed by flow cytometry (CytoFlex, BD Biosciences, USA). To inhibit caspase activity, the pan-caspase inhibitor, tripeptide fluoromethyl ketone (FMK)-derivative inhibitors Z-VAD-FMK (100 µM) (Beyotime Biotechnology, Shanghai, China), was added into cell medium for 30 min, prior to the addition of mNK-sEV (100 µg/mL). Either Annexin V or 7-AAD positive cells were counted as apoptotic cells, further divided by the total cells tested to obtain the percentage of apoptotic cells. The results were analyzed using Flowjo v10 software (Tree Star, USA).

### Cellular uptake assay

The cellular uptake assay was performed using both flow cytometry and confocal microscopy. The fluorescent dye Dio (5 µM) (Invitrogen, USA) was added to mNK-sEV suspensions and incubated for 30 min at 37 ℃. Samples were washed twice with PBS using 100 kDa MWCO at the speed of 2000*g* for 30 min at 4 ℃. 5 µg/mL Dio-labeled mNK-sEV were individually incubated with MGC803 cells for 3, 6, 12 and 24 h at 37 °C. Macropinocytosis inhibitor ethylisopropylamiloride (EIPA) (100 µM) (Sigma, USA) was added into cell medium for 30 min, prior to the addition of mNK-sEV (100 µg/mL). After each incubation, the uptake of mNK-sEV by MGC803 cells was examined by flow cytometry. For confocal microscopy, tumor cells were harvested at 10 h, washed twice with PBS and fixed in 4% paraformaldehyde. The samples were stained with Hoechst33342 (1:500) (Sigma, USA) before PBS washes and observed by confocal laser microscopy (LSM 5 Exciter, Carl Zeiss, Germany).

### Western blot analysis

Proteins were separated using 10% sodium dodecyl sulfate–polyacrylamide gel electrophoresis (SDS-PAGE) and then transferred to polyvinylidene fluoride (PVDF) membranes (Millipore, USA). Membranes were probed with specific primary antibodies and then with peroxidase-conjugated secondary antibodies. Protein bands were visualized with an enhanced chemiluminescence kit (Thermo Fisher Scientific, USA). Antibodies against the following proteins were used: cleaved caspase-3, cleaved caspase-8, cleaved polyadenosine diphosphoribose polymerase (PARP), cytochrome c (1:1000) (CST, USA) and GAPDH (1:2000) (Abcam, USA).)

### Detection of mitochondrial membrane potential

The mitochondrial membrane potential of tumor cells was examined by JC-1 probe. Cells were seeded in six-well plates (1 × 10^6^ cells per well) and incubated with mNK-sEV (100 µg/mL) for 24 h. Then, cells were treated with JC-1 probe (1:200) (Beyotime, China) for 20 min at 37 °C. After washing with ice cold PBS, the fluorescence of each sample was measured by an inverted fluorescence microscope (Olympus, Japan).

### In vivo animal experiments

5 × 10^6^ MGC803 cells or A549 cells in 200 µL PBS solutions were subcutaneously injected into the right flank of each nude mouse (n = 5, i.e., 5 mice per experimental condition). Tumor volumes and weights were assessed every three days, and tumor volumes were calculated as *V* = 0.5 × *a* × *b*^2^, where *V* = the tumor volume, *a* = longitudinal diameter, and *b* = latitudinal diameter. Mice were randomly divided into different groups when the tumor volumes reached ~ 50 mm^3^. conNK-sEV, mNK-sEV or PBS was intravenously (5 mg sEV in protein concentration per kg mouse body weight in 200 µL solution) injected into BALB/c nude mice every three days for six cycles. The body weights and survival rates of each group were monitored during treatment. All mice were sacrificed when the tumor volume of PBS group reached ~ 1000 mm^3^. At end of treatment, mouse tissue (heart, liver, spleen, lung, kidney, and tumor) were harvested, and mass of each tissue was measured. For histological analysis, all the tissue sections were fixed within 10% formalin in buffers. Then, paraffin-embedded continuous sections (4 µm thickness) were stained with hematoxylin and eosin (H&E) staining to examine the pathological changes. Images of tissue sections were examined by fully automatic digital slice scanner (Pannoramic MIDI, 3DHISTECH, Hungary).

### In vivo biodistribution of conNK-sEV or mNK-sEV

In MGC803 cells-xenografted mouse model, in vivo biodistributions of conNK-sEV and mNK-sEV were assessed and compared. First, a total of 6.4 mg/mL mNK-sEV or conk-sEV were incubated with 0.5 mg/mL of lead sulfide (PbS) quantum dots (PbS QDs) (Nirmidas Biotech, Mountain View, USA) at 37 °C, and the mixture was transferred to the ultrafiltration tube with 100 kDa MWCO to be centrifuged under 1500×*g* for 30 min, followed by PBS washing three times. Finally, the obtained sEV were suspended in 200 µL PBS and passed through a 0.45 µm polyethersulfone filter for next use. Then, PbS-labeled conNK-sEV or mNK-sEV were injected via the tail vein, and images were acquired at different time points (0.5, 1, 2, 4, 8, 12, 24, 48 h post injection or p.i.). At the end of experiments, mice were sacrificed, and major organs were extracted and measured by DeepVision NIR-II in vivo imaging system (λ_ex_ = 808 nm; λ_em_ = 1300 nm). ImageJ software 1.8.0 (National Institutes of Health, USA) was used for image assessment.

### Immunohistochemistry

For immunohistochemical analyses, all the tumor tissues were embedded in paraffin and dissected into 4 µm-thick sections. To assess the proliferation of tumor cells, tissue sections were incubated with primary monoclonal antibody against Ki-67 (1:200) (CST, USA) overnight after blocking with bovine serum albumin for 1 h. Then, tissue sections were incubated with horseradish peroxidase-conjugated secondary antibodies using a diaminobenzidine substrate kit (Vazyme, China). The histology of tumor tissues was examined under an optical microscope (Nikon, Japan).

### TUNEL staining

To assess the apoptosis of tumor cells, tumor sections were stained with TdT-mediated dUTP Nick-End Labeling (TUNEL) staining reagents according to the manufacturer’s instructions. Briefly, each tumor slice was covered with TUNEL reaction mixture (50 µL) for 60 min at 37 °C, followed by PBS washing three times. The cell nuclei were stained with Hoechst dyes. Images were taken by fluorescence microscopy (Olympus, Japan).

### RNA extraction, RT-PCR, and real-time RT-PCR

Total RNA was extracted from NK cells using Trizol Reagent (Invitrogen, USA) according to the manufacturer’s instructions, and equal amounts of RNA were used for RT-PCR and real-time RT-PCR analyses. GAPDH was used as an internal control. The sequences of specific primers are listed in Supplementary Table 1.

### Antibody blocking assay using granulysin neutralizing antibodies

To explore the mechanism behind cytotoxicity of mNK-sEV against tumors, neutralizing antibodies of GNLY (Biolegend, USA) or mouse IgG1 (mIgG1) isotype control (Biolegend, USA) were used. Tumor cells were previously incubated with anti-GNLY mAb (0.5 mg/mL) for 2 h at 37 °C and then incubated with mNK-sEV for 24 h. Apoptosis of tumor cells was assessed by flow cytometry, and key proteins in caspase pathway were examined by Western blotting.

### Statistical analysis

Data are expressed as mean ± standard deviation (SD). Differences between two groups were analyzed using a student's *t* test. Differences among three or more groups were analyzed by one-way ANOVA, followed by Tukey’s multiple comparison test. The Kruskal–Wallis H test was used to analyze the differences between in vivo tumor growths. The survival analysis was calculated by the Kaplan–Meier method and the log-rank test. All statistical analyses were performed using GraphPad Prism 8 (GraphPad Software, USA). *p* < 0.05 was considered statistically significant.

## Results

### Characterization of sEV from mNK cells

**Fig. 1:**
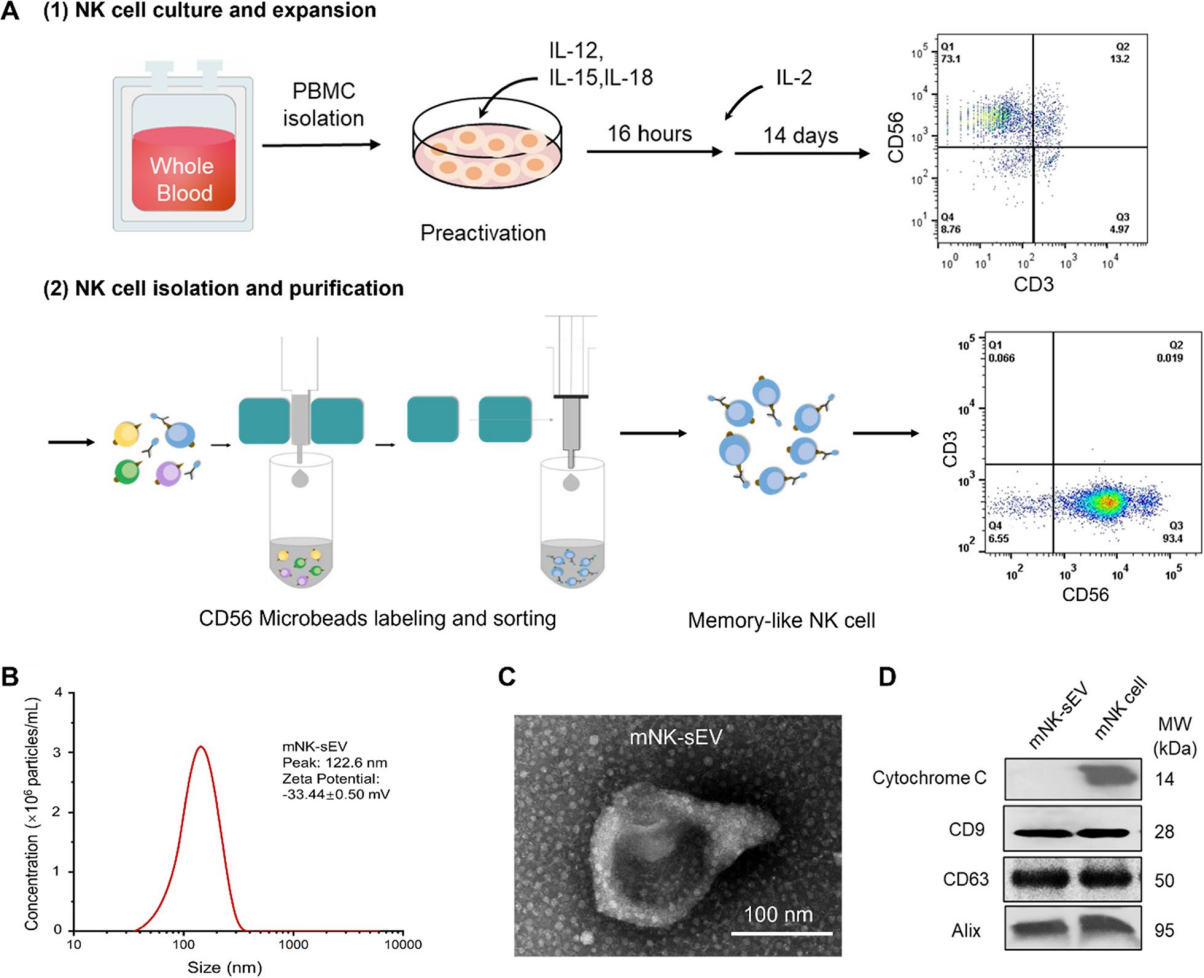
Characterization of mNK-sEV. **A** Flow chart depicting the production of mNK cells. **B** The size distribution of mNK-sEV analyzed by NTA. **C** Visualization of mNK-sEV by TEM. **D** Western blot analysis of CD9, CD63, Alix, and cytochrome c expression in NK cell lysates and mNK-sEV

We first isolated PBMCs from healthy donors and primed them with IL-12, IL-15, and IL-18 to induce the expansion and activation of NK cells in 2 weeks. On day 14, the cell culture contained > 70% activated cytokine-induced mNK cells (CD3^−^CD56^+^). After being further isolated, the purity of mNK cells was detected > 90% (CD3^−^CD56^+^) (Fig. 1A). Then mNK cells were cultured in L15 medium with 10% EV-depleted FBS for two days to secret sEV. NTA results indicated a particle distribution of mNK-sEV peaked at ~ 122 nm in diameter (Fig. 1B), with zeta potential of − 33.4 ± 0.5 mV, confirming a negatively charged membrane structure at nano scale. Characterization of mNK-sEV by TEM revealed the characteristic disc or cup shape with intact membrane structure and corroborated the particle sizes of ~ 120 nm (Fig. 1C). Furthermore, Western blot results illustrated that the biomarkers of sEV (CD9, CD63 and Alix) were present in both mNK cells and mNK-sEV, but cytochrome c, a cellular biomarker, was only displayed in mNK cells (not in mNK-sEV) (Fig. 1D). Collectively, these results verified the successful purification of mNK-sEV.

### Cytotoxicity of mNK-sEV

**Fig. 2:**
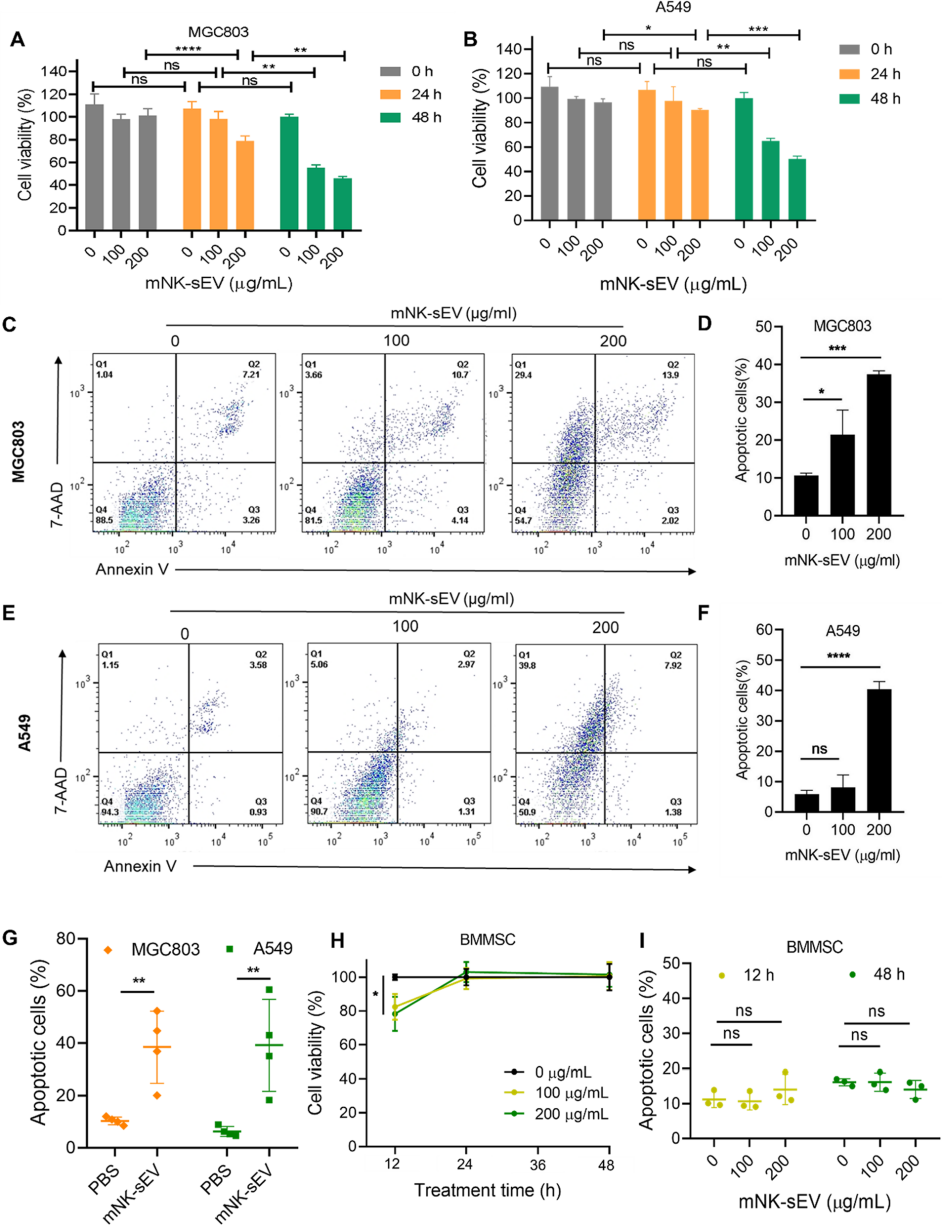
Cytotoxicity of mNK-sEV. **A**, **B** and **H** CCK-8 assays for the cytotoxicity of mNK-sEV on **A** MGC803, **B** A549, and **H** BMMSC cells, at various time points, n = 3. **C**, **E** Representative flow cytometry dot plots for cell apoptosis in **C** MGC803 and **E** A549 treated with mNK-sEV (100 and 200 µg/mL) for 24 h. 7-AAD, 7-amino-actinomycin D. **D**, **F** The percentage of apoptotic cells in **D** MGC803 and **F** A549 cells were analyzed by flow cytometry, n = 3. **G** Statistical analysis of cell apoptosis in MGC803 and A549 treated with mNK-sEV (200 µg/mL) from different donors (n = 4). **I** The percentage of apoptotic cells in BMMSC cells were analyzed by flow cytometry, n = 3. ns: no significance. *p < 0.05, ***p < 0.01, ****p < 0.0001

We next measured the cytotoxicity of mNK-sEV against human cancer cell lines, including gastric cancer cells (MGC803) and non-small cell lung cancer cells (A549). Different concentrations of mNK-sEV were incubated with cancer cells and apoptosis assays were performed to quantify the cytotoxicity. As presented in Fig. 2A and B, cell viability decreased as the concentration of mNK-sEV was increased or the incubation time was prolonged. Compared to that in the untreated group, the proportion of apoptotic cancer cells was higher in mNK-sEV treated group (Fig. 2C–F). These results were also demonstrated in mNK-sEV derived from different donors (Fig. 2G). Moreover, 200 µg/mL mNK-sEV induced more apoptosis than 100 µg/mL mNK-sEV within 24 h incubation (Fig. 2C–F). Similar results were shown in pancreatic cancer (Patu8988t) and leukemia (K562) cell lines (Figure S1). These results demonstrated that mNK-sEV treatment led to cell death in an array of cancer cell lines and this cytotoxicity was dose-dependent and time-dependent.

To examine the effect of mNK-sEV on normal cells, we isolated and cultured human bone marrow mesenchymal stem cells (BMMSCs). After identification of BMMSCs (Figure S2), we incubated mNK-sEV with BMMSCs for 12, 24 and 48 h, respectively. CCK-8 assays were then performed, and results showed that the viability of BMMSCs declined at 12 h treatment but was recovered at 48 h (Fig. 2H). However, there was no statistical difference on BMMSCs apoptosis after incubation with mNK-sEV for 12 and 48 h (Fig. 2I). These results delineated that the suppression on BMMSC viability was transient and might not be induced by cell apoptosis. Human renal tubular epithelial (HK-2) cells were also incubated with mNK-sEV to evaluate their cytotoxicity. Both CCK-8 and apoptotic assays exhibited no noticeable toxicity of the mNK-sEV on HK-2 cells after 48 h incubation (Figures S3). Put together, mNK-sEV exerted minimal toxicity on normal cells.

### mNK-sEV inhibited tumor growth in vivo

**Fig. 3:**
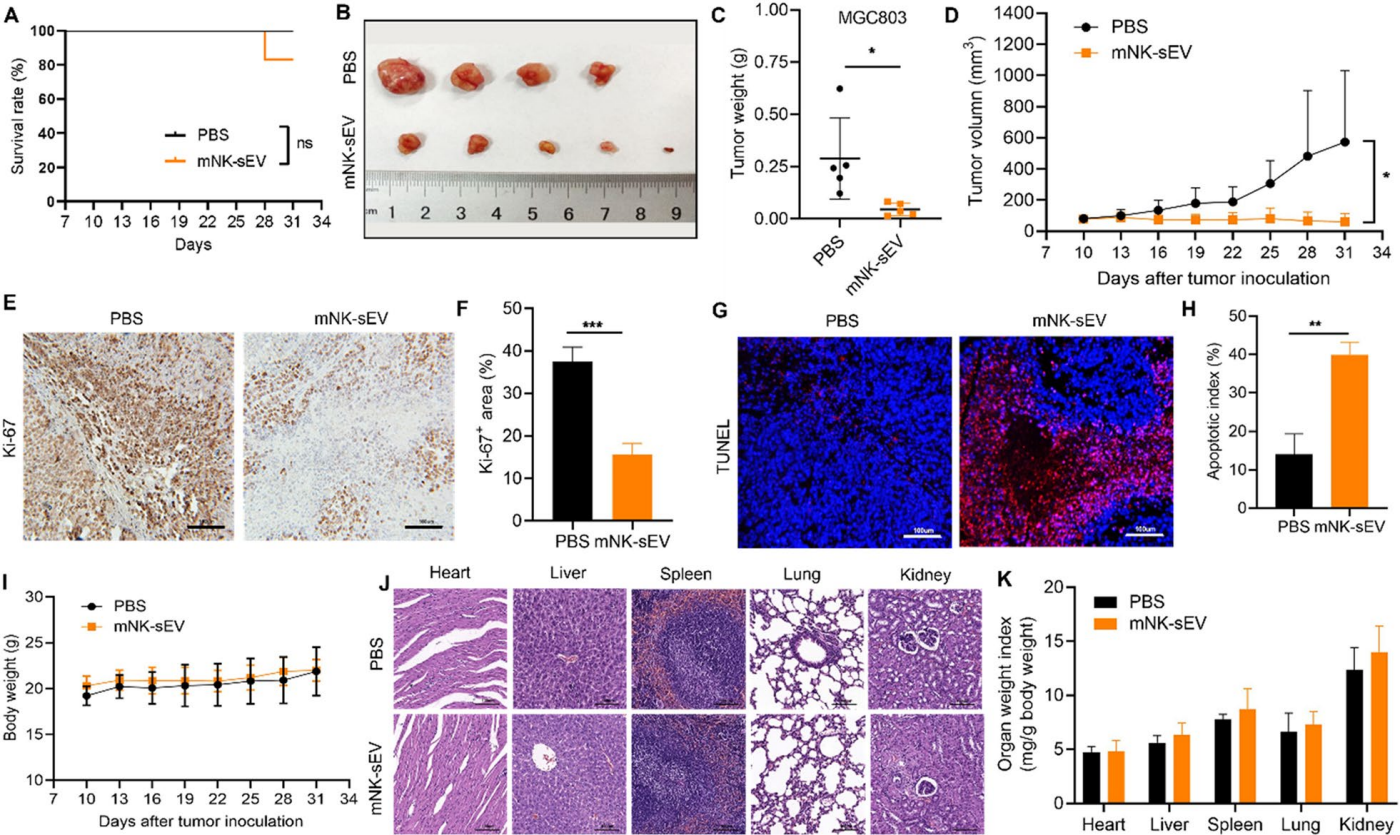
Antitumor activity of mNK-sEV in vivo. **A** Survival rates of MGC803 xenografted tumor-bearing mice that received different treatments as indicated. **B** Representative images of subcutaneous xenografted murine model established by MGC803 cells in BALB/c nude mice (n = 5 per experimental condition) that received PBS or mNK-sEV treatment. **C** Weights of the harvested tumors. **D** Tumor growth curves subject to different treatments. **E**, **F** Representative image of Ki-67 staining of tumors (scale bar = 100 µm). **G**, **H** TUNEL staining of tumors (scale bars = 100 µm). **I** Body weights of mice during in vivo efficacy study. The body weights of mice were measured every three days. Data are shown as mean ± SD. **J** Histological images of major organs (scale bar = 100 µm). **K** Weight index of major organs, including kidneys, livers, brains, lungs, and hearts from mice, which were calculated as organ weight (mg) per gram (g) of mouse body weight. *p < 0.05, **p < 0.01, ***p < 0.001

We next evaluate the antitumor effect of mNK-sEV in vivo. MGC803 cells were subcutaneously injected into BALB/c nude mice to construct a xenograft tumor model. When the tumor volume reached ~ 50 mm^3^ (n = 5 per experimental condition), mice were randomly divided into two groups and treated with PBS or mNK-sEV (5 mg sEV in protein concentration per kg mouse body weight) via tail vein injection every three days for six cycles. As a result, mNK-sEV did not significantly improve the survival rate of mice (Fig. 3A) but did substantially inhibit the tumor growth (Fig. 3B–D). Ki-67 and TUNEL staining showed that mNK-sEV suppressed cell proliferation and induced apoptosis in tumors (Fig. 3E–H). To evaluate the biosafety of mNK-sEV, we monitored the body weights of mice during mNK-sEV treatment, which showed no significant difference when compared to PBS control (Fig. 3I). Major organs were collected after mice were sacrificed, and the staining images showed that mNK-sEV treatment did not induce noticeable physiological changes in the heart, liver, spleen, lung, and kidney tissues (Fig. 3J). Organ weight normalized to mouse body weight was also comparable between PBS and mNK-sEV treatment (Fig. 3K).

Similarly, we next constructed another xenograft tumor model using A549 cells. As mirrored by tumor weight and volume (Figure S4A–4D), mNK-sEV remarkably inhibited tumor growth. Furthermore, Ki-67 and TUNEL staining showed decreased Ki-67^+^ cells and increased apoptotic cells in mNK-sEV group than in PBS group (Figure S4E–4H). In addition, mouse body weight, physiological morphology of main organs, and organ weight index showed no significant difference between mNK-sEV and PBS treatment (Figure S4I, 4J). Overall, these results pointed out that mNK-sEV had efficient antitumor activity with minimal adverse effect in vivo.

### Internalization of mNK-sEV by tumor cells

**Fig. 4:**
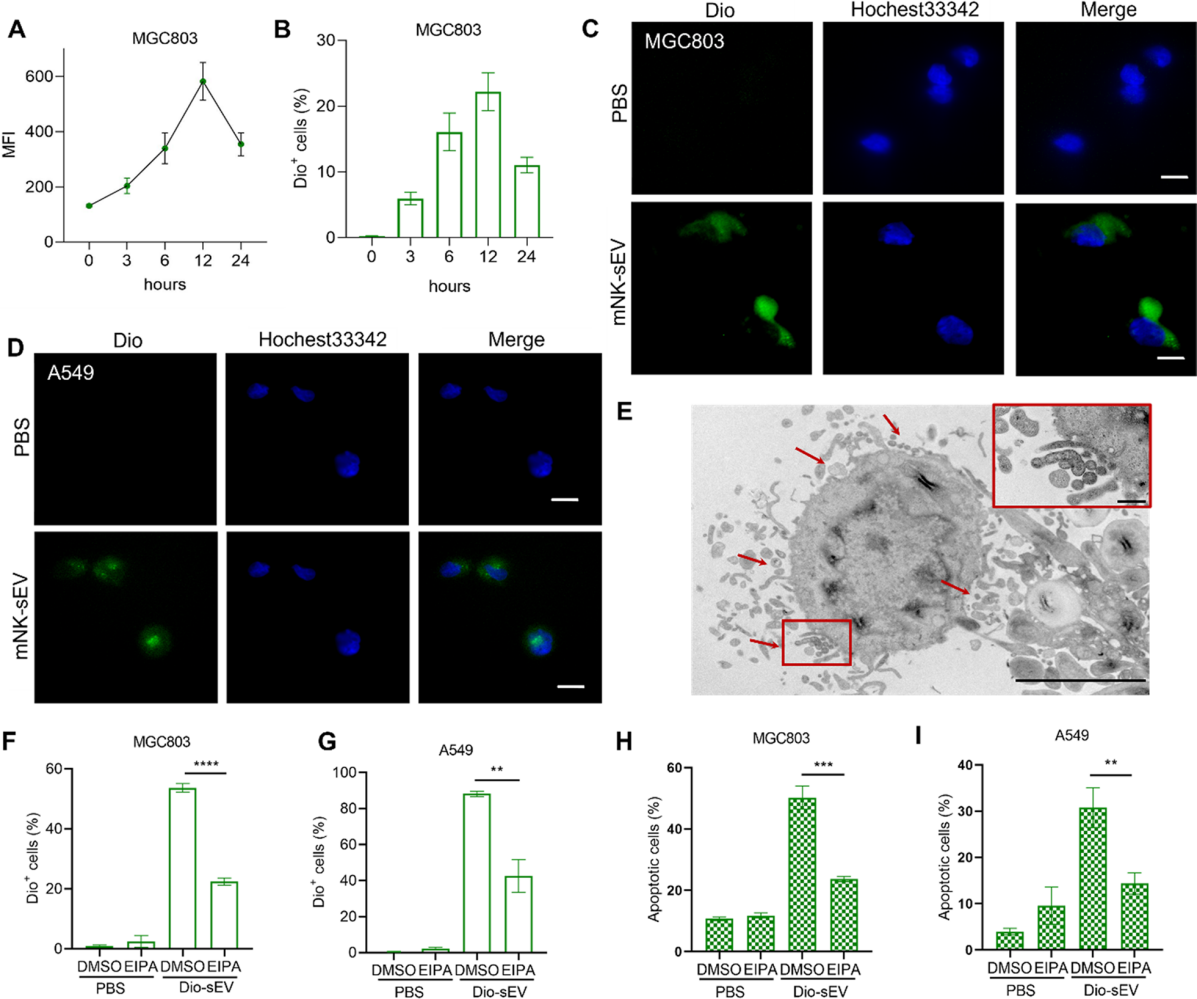
mNK-sEV uptake by tumor cells. **A**, **B** The assessment of mNK-sEV uptake by flow cytometry. Fluorescence intensities of Dio^+^ **A** MGC803 and **B** A549 cells were shown as MFI (n = 3). MFI, mean fluorescence intensity. **C**, **D** Confocal microscopy images indicated mNK-sEV internalization by **C** MGC803 and **D** A549 cells at 10 h. Cells, stained with Hochest33342 (blue), were incubated with Dio-labelled mNK-sEV (green) and their internalization was evaluated (scale bar = 5 µm). **E** Representative TEM image of mNK-sEV internalization by MGC803 cells (scale bar = 10 µm) with an insert image showing the amplified area of interest (scale bar = 100 nm). **F**, **G** Cells were pre-incubated with DMSO or 100 µM EIPA for 30 min. The percentages of Dio^+^ **F** MGC803 and **G** A549 cells were analyzed by flow cytometry (n = 3). **H**, **I** Cells were pre-incubated with DMSO or 100 µM EIPA for 30 min. The percentages of apoptotic **H** MGC803 and **I** A549 cells were analyzed by flow cytometry (n = 3). **p < 0.01, ***p < 0.001, ****p < 0.0001

To investigate the cell entry mechanism of mNK-sEV, MGC803 and A549 cells were treated with Dio-labeled mNK-sEV for 3, 6, 12, and 24 h, before detection by flow cytometry. mNK-sEV were continuously taken up by recipient cells, while Dio^+^ cells reached a peak at 12 h after treatment (Fig. 4A and B). Confocal microscopy images also showed that Dio-labeled mNK-sEV were effectively internalized by MGC803 and A549 cells (Fig. 4C and D). To further investigate the exact pathway of mNK-sEV during cellular uptake, we incubated MGC803 cells with mNK-sEV and prepared samples for TEM visualization. TEM image revealed the cytoplasmic membrane of MGC803 cells was invaginated, displaying typical endocytic process (Fig. 4E). We then incubated cancer cells with Dio-labeled mNK-sEV in the presence of EIPA, a specific inhibitor of macropinocytosis, and found out that the percentage of Dio^+^ cells was significantly reduced in EIPA treated group (Fig. 4F and G). Simultaneously, the percentage of apoptotic cells plummeted in both MGC803 and A549 cells with EIPA present (Fig. 4H and I, Figure S5). These results showed that mNK-sEV were engulfed by tumor cells via macropinocytosis, leading to apoptotic cell death.

### Apoptotic signaling pathway in cancer cells activated by mNK-sEV

**Fig. 5:**
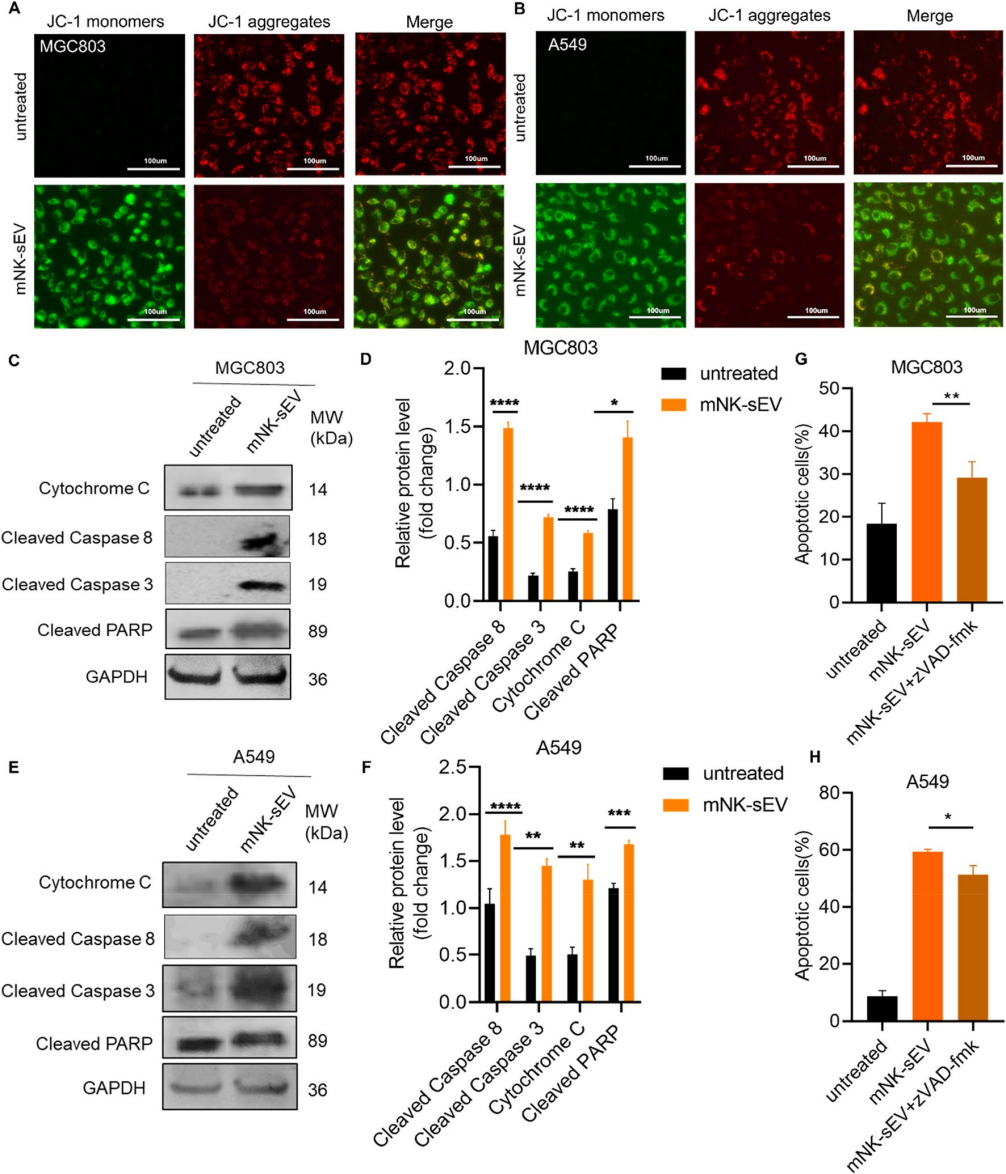
mNK-sEV induced apoptosis in tumor cells. **A**, **B** JC-1 probe staining for detecting the mitochondrial membrane potential of **A** MGC803 and **B** A549 cells when treated with mNK-sEV for 24 h. **C**–**F** Western blot assays for the expression of proteins in apoptosis signaling pathway in **C**, **D** MGC803 and **E**, **F** A549 cells when treated with 100 µg/mL mNK-sEV for 24 h. **G**, **H** Cells were incubated with 100 µM zVAD-fmk for 30 min, followed by incubation with PBS or 100 µg/mL mNK-sEV for 24 h. The percentages of apoptotic cells in **G** MGC803 and **H** A549 cells were analyzed by flow cytometry (n = 3). *p < 0.05, **p < 0.01, ****p < 0.0001

We next assessed the early apoptosis in cancer cells induced by mNK-sEV treatment using JC-1 staining analysis. It was noted that the mitochondrial membrane potential of cancer cells was considerably decreased upon mNK-sEV treatment (Fig. 5A and B), which indicated apoptosis of cancer cells. Given the fact that mNK-sEV may induce apoptosis in cancer cells, we examined apoptosis-related proteins by Western blot analysis. As shown in Fig. 5C and D, protein expressions of cytochrome c, cleaved caspase 8, cleaved caspase 3, and cleaved PARP in MGC803 cells were upregulated after 24 h mNK-sEV treatment. Similar results were obtained in A549 cells (Fig. 5E and F). Moreover, we treated cancer cells with a pan-caspase inhibitor, zVAD-fmk, prior to the addition of mNK-sEV, and found out that this pan-caspase inhibitor significantly reduced the proportion of apoptotic cells (Fig. 5G and H, Figure S6). Taken together, these results suggested that mNK-sEV might induce the apoptosis by activating caspase-dependent cell death pathway in cancer cells.

### The antitumor effects of sEV from different NK cells

**Fig. 6:**
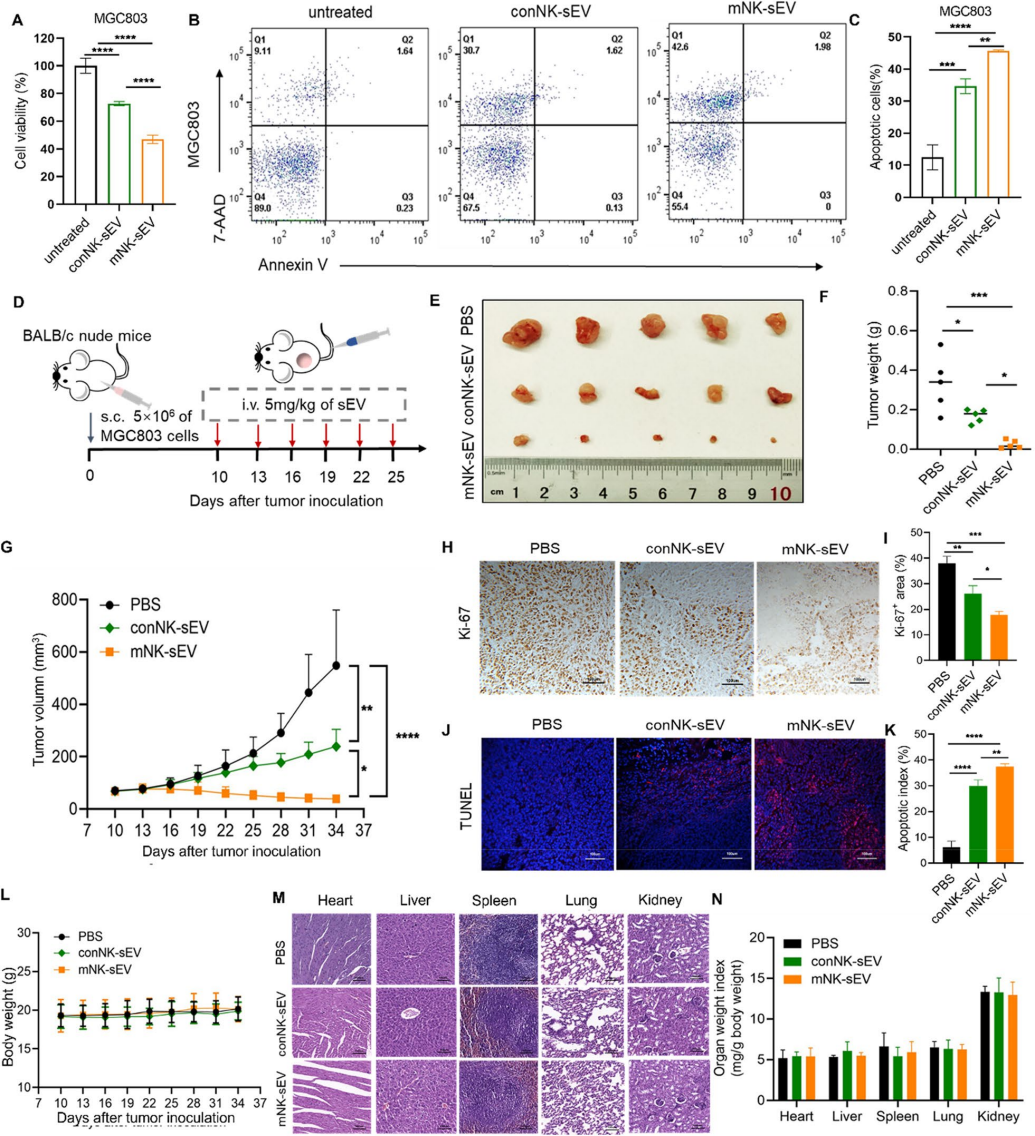
Antitumor effects of conNK-sEV and mNK-sEV. **A** CCK-8 assays for the cytotoxicity of conNK-sEV and mNK-sEV (100 µg/mL) on MGC803 cells at 24 h (n = 3). **B** Representative flow cytometry dot plot for apoptosis in MGC803 cells treated with conNK-sEV and mNK-sEV (100 µg/mL) for 24 h, respectively. **C** The percentage of apoptotic cells in MGC803 cells were analyzed by flow cytometry (n = 3). **D** Schematic design for sEV treatment in subcutaneous MGC803 cells xenografted tumor model in BALB/c nude mice. i.v., intravenous; s.c., subcutaneous. **E** Representative images of subcutaneous xenograft tumors established by MGC803 cells in BALB/c nude mice (n = 5) that were treated by PBS, conNK-sEV and mNK-sEV, respectively. **F** Weights of the harvested tumors. **G** Tumor growth curves subject to different treatments. **H**, **I** Representative image of Ki-67 staining of tumors (scale bar = 100 µm). **J**, **K** TUNEL staining of tumors (scale bar = 100 µm). **L** The body weights of mice during in vivo efficacy study were measured every 3 days. Data are shown as mean ± SD. **M** Histological images of major organs (scale bar = 100 µm). **N** Weight indices of major organ, including kidneys, livers, brains, lungs, and hearts, calculated as organ weight (mg) per gram (g) of mouse body weight. *p < 0.05, **p < 0.01, ***p < 0.001, ****p < 0.0001

We isolated PBMCs from the same healthy donor, and cultured either with single cytokine IL-2 to produce control NK (conNK) cells, or with three cytokines (IL-12, IL-15, and IL-18) to produce mNK cells (Figure S7A). During the 14-day culture, the expansion of mNK cells became much faster than that of conNK cells (Figure S7B). Compared with conNK cells, mNK cells were identified higher expression of CD25 and NKp46 and lower expression KIR2D (Figure S7C–S7E). Two types of NK cells were individually co-cultured with K562 cells (with high sensitivity to NK cell killing) at various cell ratios and tested their cytotoxicity and IFN-γ secretion. As shown in Figure S7F- S7H, mNK cells had higher cytotoxicity and secreted more IFN-γ than conNK cells. We then isolated sEV from conNK cells (conNK-sEV) and identified their physicochemical and biological features (Figure S8). At the same protein concentrations, mNK-sEV and conNK-sEV were incubated with MGC803 cells for 24 h, respectively, where mNK-sEV treatment lowered more cell viability by triggering more apoptosis than conNK-sEV (Fig. 6A–C). Similar results were observed in A549, Patu8988t and K562 cells (Figure S9).

Next we treated tumor-bearing mice with mNK-sEV and conNK-sEV, respectively, via moue tail vein injection, in the MGC803 cells-xenografted murine model (Fig. 6D). As shown in Fig. 6E–G, mNK-sEV inhibited tumor growth in a more efficient manner than did conNK-sEV. Ki-67 and TUNEL staining demonstrated that both conNK-sEV and mNK-sEV suppressed tumor growth by initiating apoptotic cell death, whereas mNK-sEV showed higher inhibitory effect (Fig. 6H–K). For biosafety evaluation, the mouse body weights during treatment were monitored, showing no noticeable difference from that in the PBS group (Fig. 6L). Pathological examination and organ weight index also exhibited no statistical difference among PBS, conNK-sEV, and mNK-sEV groups (Fig. 6M and N). These results revealed that the antitumor activity of mNK-sEV was superior to that of conNK-sEV, albeit both types of sEV showed effective anticancer effect and excellent biocompatibility.

### Comparison of in vivo pharmacokinetics, biodistribution, and tumor accumulation between conNK-sEV and mNK-sEV

We next measured in vivo pharmacokinetics, biodistribution, and tumor accumulation of both mNK-sEV and conNK-sEV in MGC803 cells-xenografted murine models. 5 mg/kg conNK-sEV or mNK-sEV labeled with fluorescent quantum dots lead sulfate (PbS) were injected intravenously into nude mice via tail vein. Mouse blood was collected at different time points, and the fluorescence intensities in various organs or tumors were determined to assess the biodistribution and tumor accumulation of PbS-labeled conNK-sEV or mNK-sEV.

**Fig. 7:**
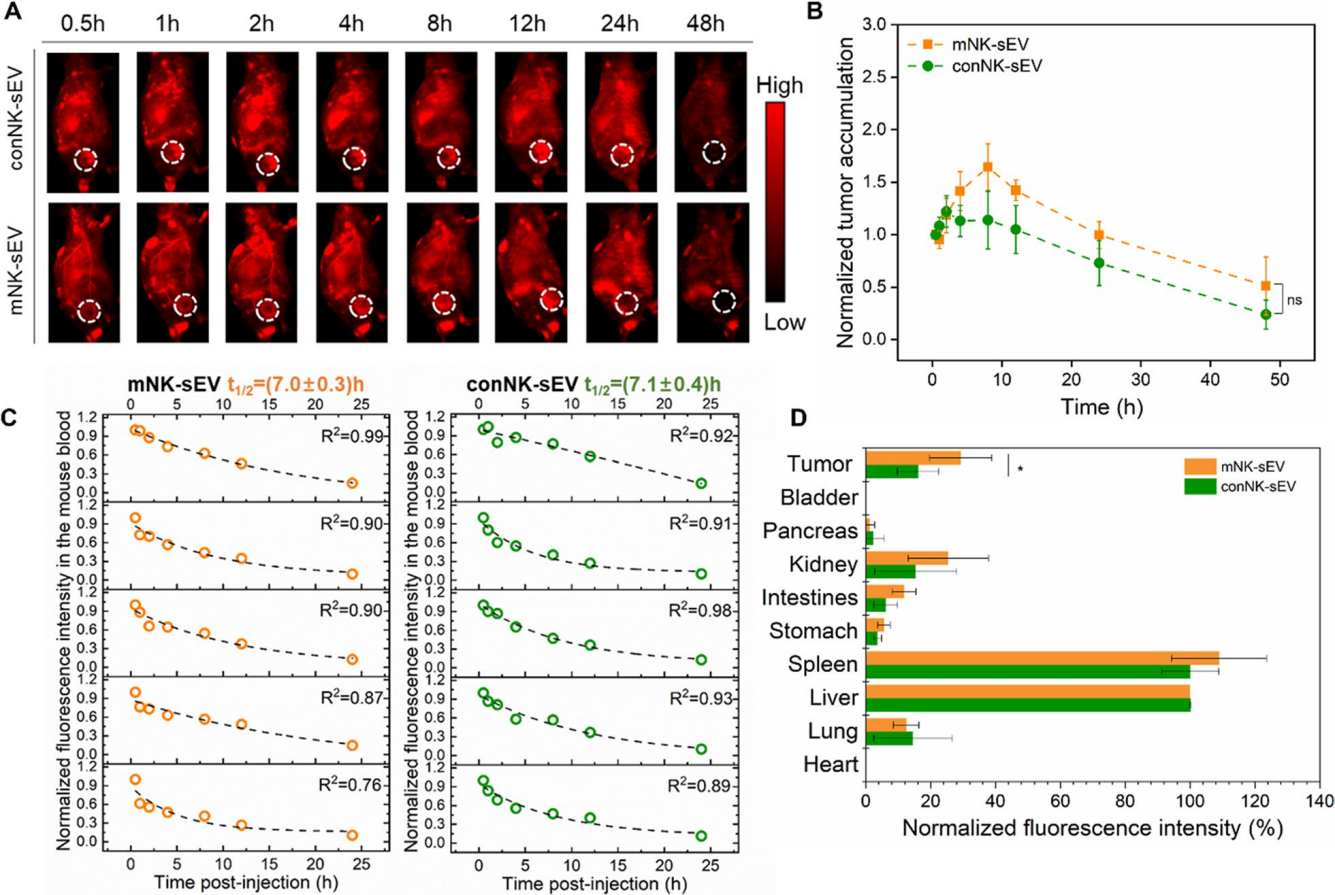
In vivo profiles of conNK-EV and mNK-EV in subcutaneous tumor models after tail vein injection. **A** Fluorescence images of one representative mouse in each condition over time, depicting the circulation of fluorescent sEV and the accumulation into the focused tumor region (white circle). **B** The fluorescence intensities of tumors were recorded over time, normalized to the first fluorescence reading in each tumor, and plotted against time (n = 5). **C** The fluorescence intensity of mouse blood was plotted versus the sampling time, fitting into an exponential function (displayed as a dotted line in each graph panel), so the exponential decay constant was acquired for conversion into the circulation half-time (*t*_1/2_). **D** At 48 h p.i., mice were sacrificed, and major organs were harvested. The fluorescence intensity of each organ was recorded per tissue area and normalized to that of liver (n = 5). ns: no significance. *p < 0.05

The NIR-II fluorescence intensities of tumors were recorded over time (Fig. 7A), being normalized to the first fluorescence reading of each tumor and plotted over time as shown in Fig. 7C. Simultaneously, mouse blood was collected at different time points post-injection (p.i.) as indicated in Fig. 7B, with the fluorescence intensity of mouse blood plotted versus the sampling time, fitting into an exponential function where the exponential decay constant was obtained and converted into the circulation half-time (*t*_1/2_). At 48 h p.i., mice were sacrificed, and the major organs were extracted. The fluorescence intensity of each organ was recorded per tissue area and normalized to that of liver (the largest organ in size with the most uptake of injection), as shown in Fig. 7D. As a result, tumor accumulation of both mNK-sEV and conNK-sEV increased and reached the peak at ~ 10 h in a similar manner. *t*_1/2_ was calculated to be 7.0 ± 0.3 h for mNK-sEV and 7.1 ± 0.4 h for conNK-sEV, showing no statistical difference (*p* > 0.05). Nevertheless, compared to conNK-sEV, mNK-sEV achieved a much higher tumor accumulation (29.2 ± 9.7 versus 16.1 ± 6.4).

### Role of granulysin in the cytotoxicity of mNK-sEV

**Fig. 8:**
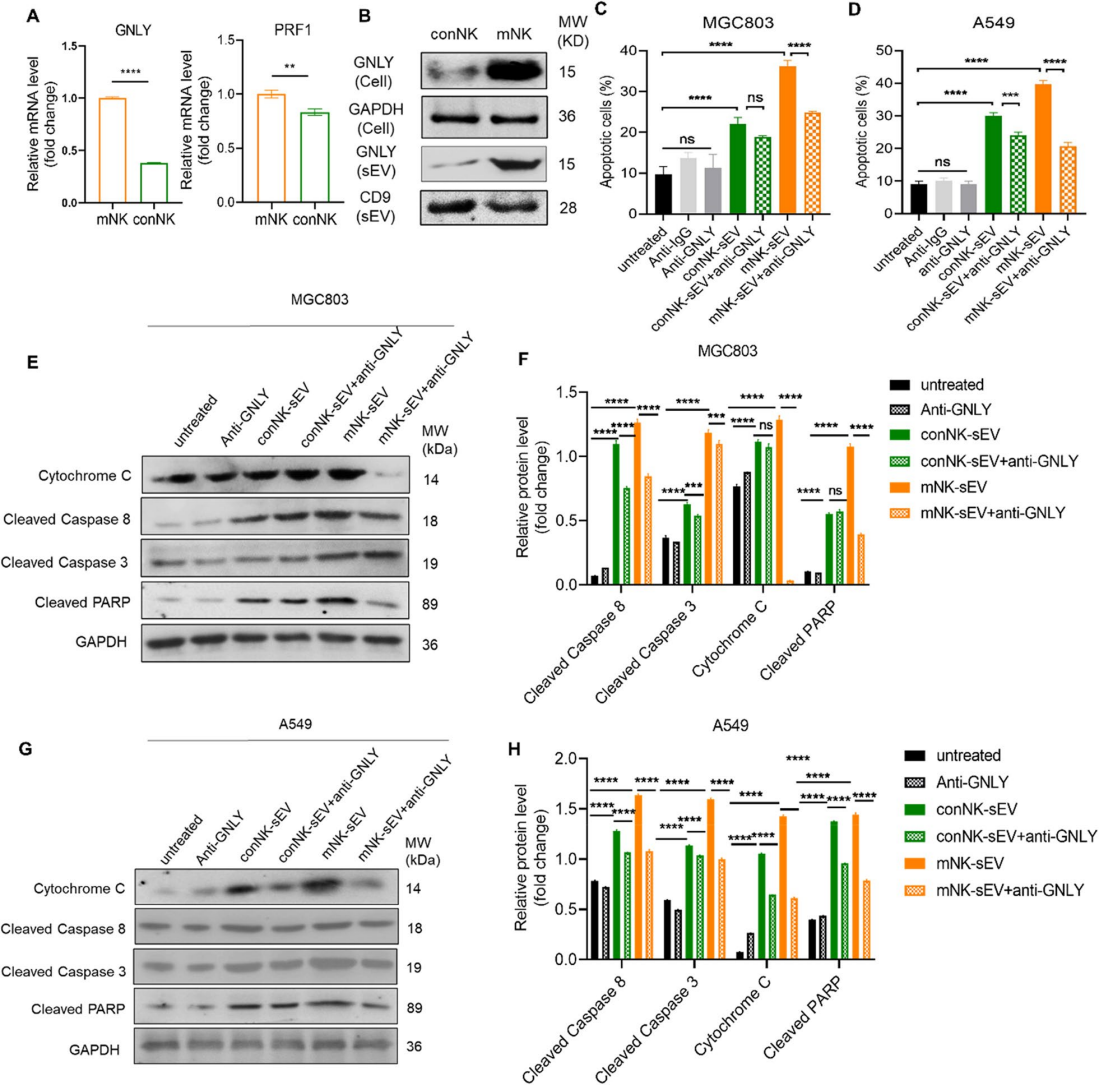
Blocking GNLY reduced cytotoxicity of mNK-sEV. **A** qRT-PCR assay for detection of cytotoxicity-related gene expression in conNK cells and mNK cells. GNLY, granulysin; PRF1, perforin 1. **B** Western blot analysis of GNLY expression in NK cells and NK cell-derived sEV. **C**, **D** Cells were incubated with 100 µg/mL of NK-sEV in the presence of GNLY neutralizing antibody. Flow cytometry analysis of cell apoptosis in **C** MGC803 and **D** A549 cells at 24 h (n = 3). **E**–**H** Western blot assays for the expression of proteins in apoptosis signaling pathway in **E** and **F** MGC803 and **G** and **H** A549 for 24 h. ns: no significance. *p < 0.05, **p < 0.01, ***p < 0.001, ****p < 0.0001

To further investigate the enhanced cytotoxicity of mNK-sEV, we performed quantitative RT-PCR to examine the expression of mRNAs encoding proteins pertinent to cytotoxic activity, including granzyme A (GZMA), granzyme B (GZMB), granzyme H (GZMH), GNLY, perforin (PRF1), Fas ligand (FASLG), and TNF-related apoptosis inducing ligand (TNFSF10) in both conNK and mNK cells (Fig. 8A and S10A). It was shown that GNLY and PRF1 were highly expressed in mNK-sEV, while GNLY mRNA expression was twice more in mNK cells than in conNK cells (Fig. 8A). Thus, we focused and speculated that GNLY could be a key mediator in mNK-sEV induced cytotoxicity. We then analyzed GNLY protein expression in both NK cells and their derived sEV. For both conNK and mNK cells, the tendency in the protein expression levels of GNLY was consistent with that in GNLY mRNA expression. Notably, based on the same protein concentration, GNLY protein was more abundant in mNK cells than conNK cells, and more in mNK-sEV than conNK-sEV (Fig. 8B).

We next explored the role of GNLY in mNK-sEV induced cytotoxicity. Cancer cells were first treated with blocking mAbs against GNLY prior to sEV treatment. Consequently, GNLY blockade resulted in the reduction of apoptosis in cancer cells. Moreover, in the presence of GNLY blocking mAb, stronger inhibition of apoptosis occurred in mNK-sEV treated cancer cells than in conNK-sEV treated cancer cells (Fig. 8C and D). Since mNK-sEV exerted their cytotoxicity by activating apoptotic pathway in cancer cells, we next examined whether GNLY plays an indispensable role in the activated apoptotic pathway by mNK-sEV treatment. To this end, caspase activity was analyzed by Western blotting in cancer cells after incubation with NK-sEV in the presence or absence of blocking antibodies against GNLY. Resultantly, blocking GNLY reduced the expression of cytochrome c, cleaved caspase 8, cleaved caspase 3, and cleaved PARP proteins in both cancer cell lines, where those expressions of apoptotic proteins were significantly lowered in cells treated with mNK-sEV compared to that treated with conNK-sEV (Fig. 8E–H). These results delineated that the enhanced cytotoxicity of mNK-EV in cancer cells was, at least in part, due to the enrichment of GNLY in mNK-EVs.

## Discussion

In this study, we first isolated sEV from mNK cells that were primed with cytokine IL-12, IL-15, and IL-18. We demonstrated that mNK-sEV were internalized into tumor cells via macropinocytosis, and exerted cytotoxicity against tumor cells by activating caspase-dependent apoptotic pathway. We also compared cytotoxicity of mNK-sEV with that of conNK-sEV, coming to a conclusion that mNK-sEV possessed an enhanced antitumor activity both in vitro and in vivo. Simultaneously, increased tumor accumulation and upregulated GNLY protein expression due to mNK-sEV treatment contributed to augmented tumor suppression.

The cytotoxicity of NK cell-derived sEV has been evaluated on a diversity of tumor cells. NK cells such as tumorigenic non-Hodgkin’s lymphoma NK92 [17] and NK92MI cells [18], non-tumorigenic NK 3.3 cell line [29], and primary human NK cells are common sources [30]. Although NK cell line-derived sEV are readily obtained, studies have shown that sEV derived from tumorigenic cells could carry cargo specific and unique to their parental cells that may negatively influence or alter recipient cells [31]. In contrast, primary NK cell-derived sEV are likely to be biologically safer and more effective for tumor treatment. Here we cultured primary mNK cells and isolated their sEV for therapeutics in murine cancer models, where mNK-sEV inhibited tumor growth by inducing apoptosis in a time-dependent and dose-dependent manner. This result stood in line with the fact that NK cells owned antitumor activity by executing cell apoptosis in cancers [32]. Meanwhile, in our murine cancer models, mNK-sEV did not improve the survival rate of mice, which may be because of the limited experimental period. And it deserved our further exploration by prolonging experimental period and monitoring survival rate of mice.

Through this study we observed that primary NK cells-derived sEV had cytotoxicity on various types of cancer cells but owned minimal impact on normal cells. This result was consistent to others [18, 29]. NK92 cell derived sEV showed no noticeable cytotoxicity on normal human kidney phoenix-A cells [18]. Although NK3.3 cell derived sEV inhibited the viability of IL-2 stimulated PBMCs at 24 h at the concentration of 25–75 µg/mL, cell viability was gradually recovered at 48–72 h [29]. Similar to these results, mNK-sEV had a transient inhibitory effect on BMMSCs at 12 h, but cell viability of BMMSCs was recovered at 24–48 h. In parallel, no cytotoxicity of mNK-sEV was found on human normal kidney HK-2 cells. Furthermore, while mNK-sEV significantly inhibited the tumor growth in xenografted murine models, no adverse effect was found on mouse body weight and major organ physiology. These findings supported that mNK-sEV were safe and effective in treating cancer cells as they remained no harm to normal cells.

Through experiments we revealed that cancer cells engulfed the Dio-labeled mNK-sEV rapidly and effectively, where the maximum uptake took place at 12 h. sEV derived from feeder cell-cultured NK cells showed the similar internalization properties to mNK-sEV [33]. Previous studies showed the size and/or surface component of sEV may affect the way of recognition and capture by recipient cells [34]. Here it was uncovered that mNK-sEV were taken up by tumor cells via macropinocytosis, a nutrient-scavenging pathway in cancers, and further activated cell apoptosis. Analysis of apoptotic pathway in tumor cells by Western blot further revealed that several key proteins, including the cleaved caspase-8, cleaved caspase-3, cleaved PARP, and cytochrome c, were upregulated in the condition of mNK-sEV treatment. Reversely, the pan-caspase inhibitor lowered the apoptotic proportion of tumor cells that were treated by mNK-sEV. Altogether, these results indicated that mNK-sEV entered tumor cells via macropinocytosis and mediated the activation of caspase-dependent apoptotic pathway.

Small EV derived from NK cells primed with different stimulation regimens demonstrated a variety of antitumor activity [19]. sEV derived from K562 cell-trained natural killer cells showed higher antitumor effect in comparison to non-trained one [35]. Dual cytokines IL-15 and IL-21 co-induced NK92-sEV had enhanced cytotoxicity on K562 cells when compared to sEV derived from single IL-15 or IL-21 cultured NK92 cells [17]. Intriguingly, we here compared cytotoxicities induced by mNK-sEV or conNK-sEV, with findings that mNK-sEV owned stronger antitumor activity in a variety of cancer cells and xenografted tumor murine models. Since IL-12, IL-15 and IL-18 can activate NK cells to prepare mNK cells [36], different combinations of cytokines may affect the characteristics of NK cells and thereby their derived sEV.

To better understand the difference in antitumor effects of sEVs derived from differently cultured NK cells, we performed fluorescence imaging in animal models to study and compare their pharmacokinetics, biodistribution and tumor accumulation. Our results demonstrated that mNK-sEV accumulated more in tumors than conNK-sEV did, which might contribute to their stronger antitumor ability. In addition, IL-12, IL-15 and IL-18 primed mNK cells showed different expression levels of proteins pertinent to cytotoxicity with conNK cells. We identified the 15 kDa GNLY precursor was highly enriched in both mNK cells and their derived mNK-sEV than that in conNK cells and conNK-sEV, respectively. As IL-15 is an activation stimulus that regulates GNLY [37, 38], we speculate that GNLY is mainly upregulated by IL-15 in mNK cells and so in their sEV. In addition, 15 kDa GNLY precursor can be truncated to its 9 kDa active form after entering tumor cells to function [17, 29, 39]. Conversely, blocking GNLY with neutralizing antibody resulted in the reduced cytotoxicity induced by mNK-sEV. Meanwhile, activated apoptosis was mitigated in mNK-sEV treatment after GNLY blocking. Therefore, we hypothesized that GNLY directly interferes in mitochondrial apoptosis pathway induced by sEV of NK cell origin, at least partially responsible for enhanced antitumor activity of mNK-sEV.

## Conclusion

In closing we here reported that sEV derived from mNK cells effectively exerted dose-dependent antitumor effect on cancer cells and murine models of human gastric and lung cancers. They were engulfed by tumor cells via macrophinocytosis, so promoting cell death by activating caspase-dependent apoptotic pathway. Comparison of mNK-sEV induced cytotoxicity to conNK-sEV one highlighted an enhanced antitumor effect of mNK-sEV. More tumor accumulation and higher GNLY expression can partially explain the greater antitumor activity of mNK-sEV. Put together, mNK-sEV represent an efficient and reliable option for cancer therapeutics and warrant further development as a potential immunotherapeutic strategy in clinical settings.

### Supplementary Information

Supplementary Material 1.

## Data Availability

The data that support the findings of this study are available from the corresponding author(s) upon reasonable request.

## Abbreviations

BCA: Bicinchoninic acid
conNK cell: Conventionally cultured natural killer cell
EIPA: Ethylisopropylamiloride
FBS: Fetal bovine serum
FASLG: Fas ligand
GNLY: Granulysin
GZMA: Granzyme A
GZMB: Granzyme B
GZMH: Granzyme H
H&E: Hematoxylin and eosin
mNK cell: Memory-like natural killer cell
NK cell: Natural killer cell
NTA: Nanosight tracking analysis
PBS: Phosphate buffered saline
PbS: Lead sulfide
PVDF: Polyvinylidene fluoride
PARP: Polyadenosine diphosphoribose polymerase
PBMCs: Peripheral blood mononuclear cells
PRF1: Perforin
sEV: Small extracellular vesicles
SDS-PAGE: Sodium dodecyl sulfate–polyacrylamide gel electrophoresis
TUNEL: TdT-mediated dUTP Nick-End Labeling
TEM: Transmission electron microscopy
TNFSF10: TNF-related apoptosis inducing ligand

## References

[1] Fang F, Xie S, Chen M, Li Y, Yue J, Ma J, Shu X, He Y, Xiao W, Tian Z. Advances in NK cell production. Cell Mol Immunol. 2022;19:460–81. 34983953 10.1038/s41423-021-00808-3 PMC8975878

[2] Deng W, Gowen BG, Zhang L, Wang L, Lau S, Iannello A, Xu J, Rovis TL, Xiong N, Raulet DH. Antitumor immunity A shed NKG2D ligand that promotes natural killer cell activation and tumor rejection. Science. 2015;348:136–9. 25745066 10.1126/science.1258867 PMC4856222

[3] Vivier E, Rebuffet L, Narni-Mancinelli E, Cornen S, Igarashi RY, Fantin VR. Natural killer cell therapies. Nature. 2024;626:727–36. 38383621 10.1038/s41586-023-06945-1

[4] Zalfa C, Paust S. Natural killer cell interactions with myeloid derived suppressor cells in the tumor microenvironment and implications for cancer immunotherapy. Front Immunol. 2021;12: 633205. 34025641 10.3389/fimmu.2021.633205 PMC8133367

[5] Zhang H, Yang L, Wang T, Li Z. NK cell-based tumor immunotherapy. Bioact Mater. 2024;31:63–86. 37601277 10.1016/j.bioactmat.2023.08.001 PMC10432724

[6] Wolf NK, Kissiov DU, Raulet DH. Roles of natural killer cells in immunity to cancer, and applications to immunotherapy. Nat Rev Immunol. 2023;23:90–105. 35637393 10.1038/s41577-022-00732-1

[7] Dagher OK, Posey AD Jr. Forks in the road for CAR T and CAR NK cell cancer therapies. Nat Immunol. 2023;24:1994–2007. 38012406 10.1038/s41590-023-01659-y PMC12798859

[8] Ghaedrahmati F, Esmaeil N, Abbaspour M. Targeting immune checkpoints: how to use natural killer cells for fighting against solid tumors. Cancer Commun. 2023;43:177–213. 10.1002/cac2.12394 PMC9926962 36585761

[9] Fu P, Guo Y, Luo Y, Mak M, Zhang J, Xu W, Qian H, Tao Z. Visualization of microRNA therapy in cancers delivered by small extracellular vesicles. J Nanobiotechnol. 2023;21:457. 10.1186/s12951-023-02187-5 PMC10685536 38031152

[10] Yang C, Xue Y, Duan Y, Mao C, Wan M. Extracellular vesicles and their engineering strategies, delivery systems, and biomedical applications. J Control Release. 2024;365:1089–123. 38065416 10.1016/j.jconrel.2023.11.057

[11] Fu P, Zhang J, Li H, Mak M, Xu W, Tao Z. Extracellular vesicles as delivery systems at nano-/micro-scale. Adv Drug Deliv Rev. 2021;179: 113910. 34358539 10.1016/j.addr.2021.113910 PMC8986465

[12] Kamerkar S, LeBleu VS, Sugimoto H, Yang S, Ruivo CF, Melo SA, Lee JJ, Kalluri R. Exosomes facilitate therapeutic targeting of oncogenic KRAS in pancreatic cancer. Nature. 2017;546:498–503. 28607485 10.1038/nature22341 PMC5538883

[13] Seo N, Shirakura Y, Tahara Y, Momose F, Harada N, Ikeda H, Akiyoshi K, Shiku H. Activated CD8+ T cell extracellular vesicles prevent tumour progression by targeting of lesional mesenchymal cells. Nat Commun. 2018;9:435. 29382847 10.1038/s41467-018-02865-1 PMC5789986

[14] Rezaie J, Feghhi M, Etemadi T. A review on exosomes application in clinical trials: perspective, questions, and challenges. Cell Commun Signal. 2022;20:145. 36123730 10.1186/s12964-022-00959-4 PMC9483361

[15] Elahi FM, Farwell DG, Nolta JA, Anderson JD. Preclinical translation of exosomes derived from mesenchymal stem/stromal cells. Stem Cells. 2020;38:15–21. 31381842 10.1002/stem.3061 PMC7004029

[16] Federici C, Shahaj E, Cecchetti S, Camerini S, Casella M, Iessi E, Camisaschi C, Paolino G, Calvieri S, Ferro S, Cova A, Squarcina P, Bertuccini L, Iosi F, Huber V, Lugini L. Natural-killer-derived extracellular vesicles: immune sensors and interactors. Front Immunol. 2020;11:262. 32231660 10.3389/fimmu.2020.00262 PMC7082405

[17] Enomoto Y, Li P, Jenkins LM, Anastasakis D, Lyons GC, Hafner M, Leonard WJ. Cytokine-enhanced cytolytic activity of exosomes from NK cells. Cancer Gene Ther. 2022;29:734–49. 34316033 10.1038/s41417-021-00352-2 PMC9209332

[18] Zhu L, Kalimuthu S, Gangadaran P, Oh JM, Lee HW, Baek SH, Jeong SY, Lee SW, Lee J, Ahn BC. Exosomes derived from natural killer cells exert therapeutic effect in melanoma. Theranostics. 2017;7:2732–45. 28819459 10.7150/thno.18752 PMC5558565

[19] Zhu L, Kalimuthu S, Oh JM, Gangadaran P, Baek SH, Jeong SY, Lee S-W, Lee J, Ahn B-C. Enhancement of antitumor potency of extracellular vesicles derived from natural killer cells by IL-15 priming. Biomaterials. 2019;190–191:38–50. 30391801 10.1016/j.biomaterials.2018.10.034

[20] Gunesch JT, Angelo LS, Mahapatra S, Deering RP, Kowalko JE, Sleiman P, Tobias JW, Monaco-Shawver L, Orange JS, Mace EM. Genome-wide analyses and functional profiling of human NK cell lines. Mol Immunol. 2019;115:64–75. 30054012 10.1016/j.molimm.2018.07.015 PMC6345623

[21] Shapiro RM, Birch GC, Hu G, Vergara Cadavid J, Nikiforow S, Baginska J, Ali AK, Tarannum M, Sheffer M, Abdulhamid YZ, Rambaldi B, Arihara Y, Reynolds C, Halpern MS, Rodig SJ, Cullen N, Wolff JO, Pfaff KL, Lane AA, Lindsley RC, Cutler CS, Antin JH, Ho VT, Koreth J, Gooptu M, Kim HT, Malmberg KJ, Wu CJ, Chen J, Soiffer RJ, Ritz J, Romee R. Expansion, persistence, and efficacy of donor memory-like NK cells infused for posttransplant relapse. J Clin Invest. 2022. 10.1172/JCI154334. 35349491 10.1172/JCI154334 PMC9151697

[22] Romee R, Schneider SE, Leong JW, Chase JM, Keppel CR, Sullivan RP, Cooper MA, Fehniger TA. Cytokine activation induces human memory-like NK cells. Blood. 2012;120:4751–60. 22983442 10.1182/blood-2012-04-419283 PMC3520618

[23] Ewen EM, Pahl JHW, Miller M, Watzl C, Cerwenka A. KIR downregulation by IL-12/15/18 unleashes human NK cells from KIR/HLA-I inhibition and enhances killing of tumor cells. Eur J Immunol. 2018;48:355–65. 29105756 10.1002/eji.201747128

[24] Carreira-Santos S, Lopez-Sejas N, Gonzalez-Sanchez M, Sanchez-Hernandez E, Pera A, Hassouneh F, Duran E, Solana R, Casado JG, Tarazona R. Enhanced expression of natural cytotoxicity receptors on cytokine-induced memory-like natural killer cells correlates with effector function. Front Immunol. 2023;14:1256404. 37908353 10.3389/fimmu.2023.1256404 PMC10613704

[25] Terren I, Orrantia A, Astarloa-Pando G, Amarilla-Irusta A, Zenarruzabeitia O, Borrego F. Cytokine-induced memory-like nk cells: from the basics to clinical applications. Front Immunol. 2022;13: 884648. 35603208 10.3389/fimmu.2022.884648 PMC9114299

[26] Dong H, Ham JD, Hu G, Xie G, Vergara J, Liang Y, Ali A, Tarannum M, Donner H, Baginska J, Abdulhamid Y, Dinh K, Soiffer RJ, Ritz J, Glimcher LH, Chen J, Romee R. Memory-like NK cells armed with a neoepitope-specific CAR exhibit potent activity against NPM1 mutated acute myeloid leukemia. Proc Natl Acad Sci USA. 2022;119: e2122379119. 35696582 10.1073/pnas.2122379119 PMC9231490

[27] Romee R, Rosario M, Berrien-Elliott MM, Wagner JA, Jewell BA, Schappe T, Leong JW, Abdel-Latif S, Schneider SE, Willey S, Neal CC, Yu L, Oh ST, Lee YS, Mulder A, Claas F, Cooper MA, Fehniger TA. Cytokine-induced memory-like natural killer cells exhibit enhanced responses against myeloid leukemia. Sci Transl Med. 2016;8: 357ra123. 27655849 10.1126/scitranslmed.aaf2341 PMC5436500

[28] Shi Y, Zhang J, Mao Z, Jiang H, Liu W, Shi H, Ji R, Xu W, Qian H, Zhang X. Extracellular vesicles from gastric cancer cells induce PD-L1 expression on neutrophils to suppress T-cell immunity. Front Oncol. 2020;10:629. 32477934 10.3389/fonc.2020.00629 PMC7237746

[29] Cochran AM, Kornbluth J. Extracellular vesicles from the human natural killer cell line NK3.3 have broad and potent anti-tumor activity. Front Cell Dev Biol. 2021;9: 698639. 34368150 10.3389/fcell.2021.698639 PMC8343581

[30] Jong AY, Wu CH, Li J, Sun J, Fabbri M, Wayne AS, Seeger RC. Large-scale isolation and cytotoxicity of extracellular vesicles derived from activated human natural killer cells. J Extracell Vesicles. 2017;6:1294368. 28326171 10.1080/20013078.2017.1294368 PMC5345580

[31] Mashouri L, Yousefi H, Aref AR, Ahadi AM, Molaei F, Alahari SK. Exosomes: composition, biogenesis, and mechanisms in cancer metastasis and drug resistance. Mol Cancer. 2019;18:75. 30940145 10.1186/s12943-019-0991-5 PMC6444571

[32] Lee HW, Singh TD, Lee SW, Ha JH, Rehemtulla A, Ahn BC, Jeon YH, Lee J. Evaluation of therapeutic effects of natural killer (NK) cell-based immunotherapy in mice using in vivo apoptosis bioimaging with a caspase-3 sensor. FASEB J. 2014;28:2932–41. 24736413 10.1096/fj.13-243014

[33] Di Pace AL, Tumino N, Besi F, Alicata C, Conti LA, Munari E, Maggi E, Vacca P, Moretta L. Characterization of human NK cell-derived exosomes: role of DNAM1 receptor in exosome-mediated cytotoxicity against tumor. Cancers. 2020;12:661. 32178479 10.3390/cancers12030661 PMC7140072

[34] Kimiz-Gebologlu I, Oncel SS. Exosomes: large-scale production, isolation, drug loading efficiency, and biodistribution and uptake. J Control Release. 2022;347:533–43. 35597405 10.1016/j.jconrel.2022.05.027

[35] Mohammadi F, Hashemi ZS, Forooshani RS, Alizadeh S. Bioactivity of exosomes derived from trained natural killer cells versus non-trained one: more functional and antitumor activity. Biomed Res Int. 2022;2022:5396628. 36060136 10.1155/2022/5396628 PMC9433262

[36] Bednarski JJ, Zimmerman C, Berrien-Elliott MM, Foltz JA, Becker-Hapak M, Neal CC, Foster M, Schappe T, McClain E, Pence PP, Desai S, Kersting-Schadek S, Wong P, Russler-Germain DA, Fisk B, Lie WR, Eisele J, Hyde S, Bhatt ST, Griffith OL, Griffith M, Petti AA, Cashen AF, Fehniger TA. Donor memory-like NK cells persist and induce remissions in pediatric patients with relapsed AML after transplant. Blood. 2022;139:1670–83. 34871371 10.1182/blood.2021013972 PMC8931511

[37] Hogg A, Huante M, Ongaya A, Williams J, Ferguson M, Cloyd M, Amukoye E, Endsley J. Activation of NK cell granulysin by mycobacteria and IL-15 is differentially affected by HIV. Tuberculosis. 2011;91(Suppl 1):S75-81. 22099421 10.1016/j.tube.2011.10.015

[38] Endsley JJ, Endsley MA, Estes DM. Bovine natural killer cells acquire cytotoxic/effector activity following activation with IL-12/15 and reduce *Mycobacterium bovis* BCG in infected macrophages. J Leukoc Biol. 2006;79:71–9. 16275895 10.1189/jlb.0505239

[39] Wu CH, Li J, Li L, Sun J, Fabbri M, Wayne AS, Seeger RC, Jong AY. Extracellular vesicles derived from natural killer cells use multiple cytotoxic proteins and killing mechanisms to target cancer cells. J Extracell Vesicles. 2019;8:1588538. 30891164 10.1080/20013078.2019.1588538 PMC6419691

