# Supplementary material for: Therapeutic effect of small extracellular vesicles from cytokine-induced memory-like natural killer cells on solid tumors
Source: J Nanobiotechnology. 2024 Jul 29;22:447. doi: 10.1186/s12951-024-02676-1 (PMC11285333; doi:10.1186/s12951-024-02676-1)
Supplement: Supplementary file 1 — Supplementary Material 1. [file 12951_2024_2676_MOESM1_ESM.docx]

Supplementary materials

for

**Therapeutic effect of small extracellular vesicles from cytokine-induced memory-like natural killer cells on solid tumors**

Yinghong Shi^1,2,†^, Yanxia Chen^1, †^, Yi Wang^3, †^, Dan Mo^3^, Huisheng Ai^3^, Jianguo Zhang^4,*^, Mei Guo^3,*^, Hui Qian^1,2,*^

^1^Jiangsu Province Key Laboratory of Medical Science and Laboratory Medicine, Department of Laboratory Medicine, School of Medicine, Jiangsu University, Zhenjiang, Jiangsu 212013, China

^2^Zhenjiang Municipal Key Laboratory of High Technology for Basic and Translational Research on Exosomes, Zhenjiang, Jiangsu 212013, China

^3^The Fifth Medical Center of Chinese PLA General Hospital, Beijing 100039, China

^4^Department of Emergency Medicine, The Affiliated Hospital, Jiangsu University, Zhenjiang, Jiangsu 212001, China

^†^Those authors contributed equally to this work.

* Correspondences should be addressed to:

Jianguo Zhang: [1000011431@ujs.edu.cn](mailto:1000011431@ujs.edu.cn)

Mei Guo: [guom196801@aliyun.com](mailto:guom196801@aliyun.com) (ORCID: 0000-0001-6954-8873)

Hui Qian: [lstmmmlst@163.com](mailto:lstmmmlst@163.com) (ORCID: 0000-0002-0098-3196)

**Supplementary methods**

**S1. BMMSC isolation and identification**

Bone marrow cells were collected in the Department of Orthopedics at the Affiliated Hospital of Jiangsu University. Informed consents were obtained from all patients, and the study was approved by the Research Ethics Committee, The Affiliated Hospital of Jiangsu University, China. Bone marrow was diluted with the equal volume of PBS. Diluted bone marrow was isolated using 1.077 g/mL Ficoll solution. The isolated cells were cultured in DMEM cell culture medium supplemented with 10% fetal bovine serum at 37°C in 5% CO_2_. Adherent cells were collected after 5 days. After culturing for 5 days, colonies of fibroblast-like cells formed. For identification of bone marrow (BM)-MSCs, the expressions of specific surface antigens CD44, CD105, CD34 and CD45 (Biolegend, USA) of BM-MSCs were detected by flow cytometry.

**S2. NK cell cytotoxicity and cytokine measurement**

Flow cytometric analysis was performed to detect cytotoxicity and expression of cytokine IFN-γ of NK cells. For analysis of cytotoxicity, conNK cells and mNK cells from the same donor were co-cultured with K562 cells with different ratios of 1:1, 3:1, 5:1 and 10:1 for 4 h, respectively. The apoptosis of K562 cells was tested using an Annexin V-APC/7-AAD Apoptosis Detection Kit (Vazyme, China). For intracellular cytokine measurement, NK cells were stimulated for 5 h with Brefeldin A /Monensin Mixture (250×) (Yishan Biotec, China). IFN-γ staining was performed after fixation and permeabilization using Perm/Wash solution (FMS, China). The results were analyzed using FlowJo_v10.0 software (BD biosciences, USA).

**Table S1. Primers used for qRT-PCR.**

| Gene | Sequence (5’-3’) | Product size (bp) | Annealing temperature (°C) |
| --- | --- | --- | --- |
| PRF1-F | GCTATCGTTAGTGCTAGTGGAT | 99 | 55 |
| PRF1-R | ATCTGTCTGATGCGTATCCAAT |  |  |
| TNFSF10-F | TGGCTGTAACTTACGTGTACTT | 129 | 55 |
| TNFSF10-R | TCATACTCTCTTCGTCATTGGG |  |  |
| GNLY-F | CTACAGGACCTGTCTGACGATA | 80 | 55 |
| GNLY-R | CAGCATTGGAAACACTTCTCTG |  |  |
| GRMH-F | CTCAATGAGCACTTTAGCAACC | 93 | 55 |
| GRMH-R | CTGTAATTGCCATGGAAGAGAC |  |  |
| GRMB-F | GAAAGTGCGAATCTGACTTACG | 157 | 55 |
| GRMB-R | TTGTTTCGTCCATAGGAGACAA |  |  |
| GRMA-F | GCGAAGGTGACCTTAAACTTTT | 177 | 55 |
| GRMA-R | TGACTTCTCTCAGAGTATCGGA |  |  |
| FASLG-F | CACAGCATCATCTTTGGAGAAG | 202 | 55 |
| FASLG-R | GTACAGCCCAGTTTCATTGATC |  |  |
| GAPDH-F | AAGGTCGGAGTCAACGGATT | 177 | 55 |
| GAPDH-R | ATCTCGCTCCTGGAAGATG |  |  |
| F, forward; R, reverse. | | | |

**Supplementary Figures**


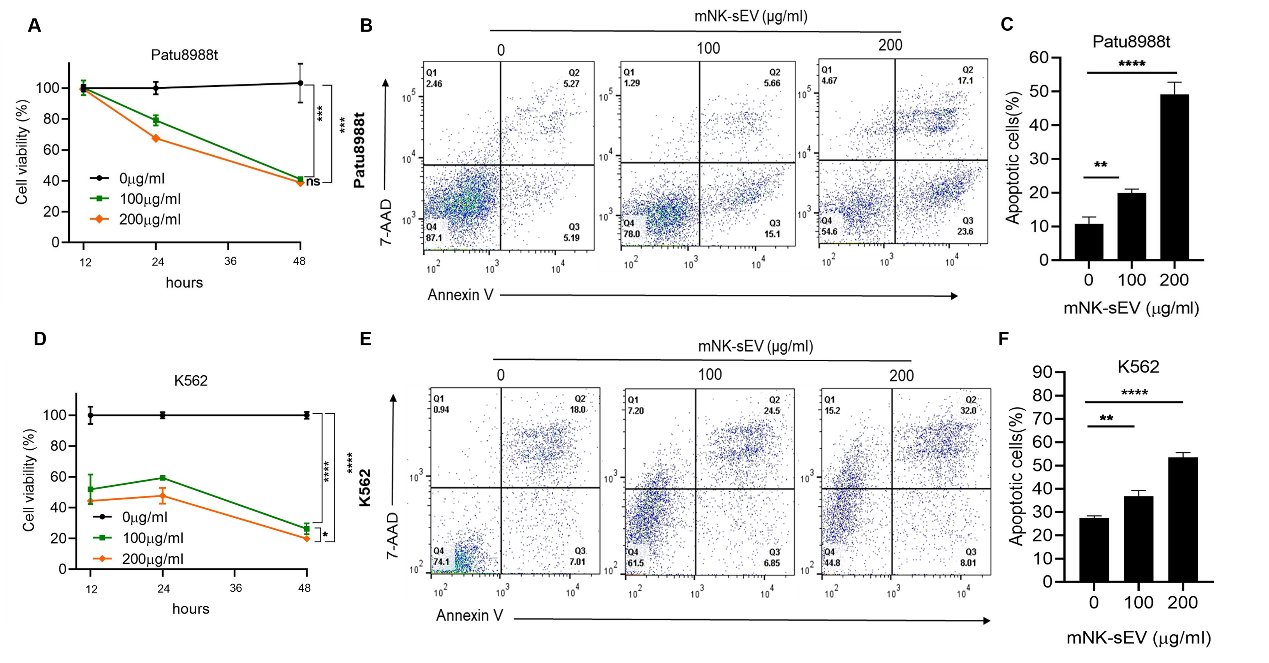


**Figure S1.** Cytotoxicity of mNK-sEV on tumor cells. (A and D) CCK-8 assays for the cytotoxicity of mNK-sEV on (A) human pancreatic cancer Patu8988t cells and (D) myeloid leukemia K562 cells at various time points (n=3). (B and E) Flow cytometry analysis of cell apoptosis in (B) pancreatic cancer Patu8988t cells and (E) myeloid leukemia K562 cells treated with mNK-sEV for 24 h. 7-AAD, 7-amino-actinomycin D. (C and F) The percentage of apoptosis in (C) Patu8988t and (F) K562 cells (n=3). ns, no significance. **p*<0.05, ***p*<0.01, ****p*< 0.001, *****p*<0.0001.

**
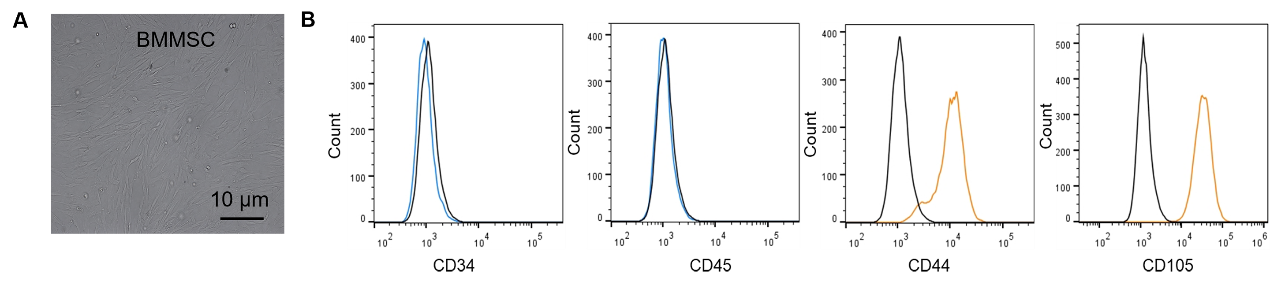
**

**Figure S2.** Characterization of human bone marrow mesenchymal stem cell BMMSCs. (A) The morphology of BMMSCs (scale bar=10 μm) indicated by light-field optical microscope; (B) The surface markers on BMMSCs by flow cytometry analysis.

**
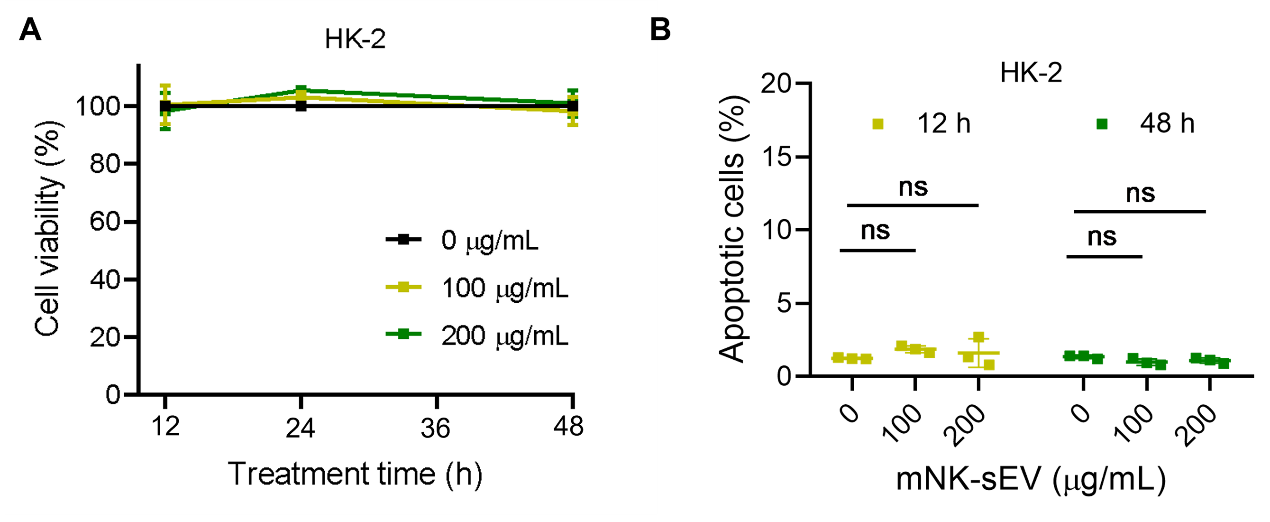
**

**Figure S3.** Cytotoxicity of mNK-sEV. (A) CCK-8 assays for the cytotoxicity of mNK-sEV on HK-2 cells at various time points (n=3). (B) Flow cytometry analysis of cell apoptosis in HK-2 cells treated with mNK-sEV for 24 h (n=3). The percentage of apoptotic cells in HK-2 cells. ns, no significance. **p*<0.05, ****p*<0.01, *****p*<0.0001.

**
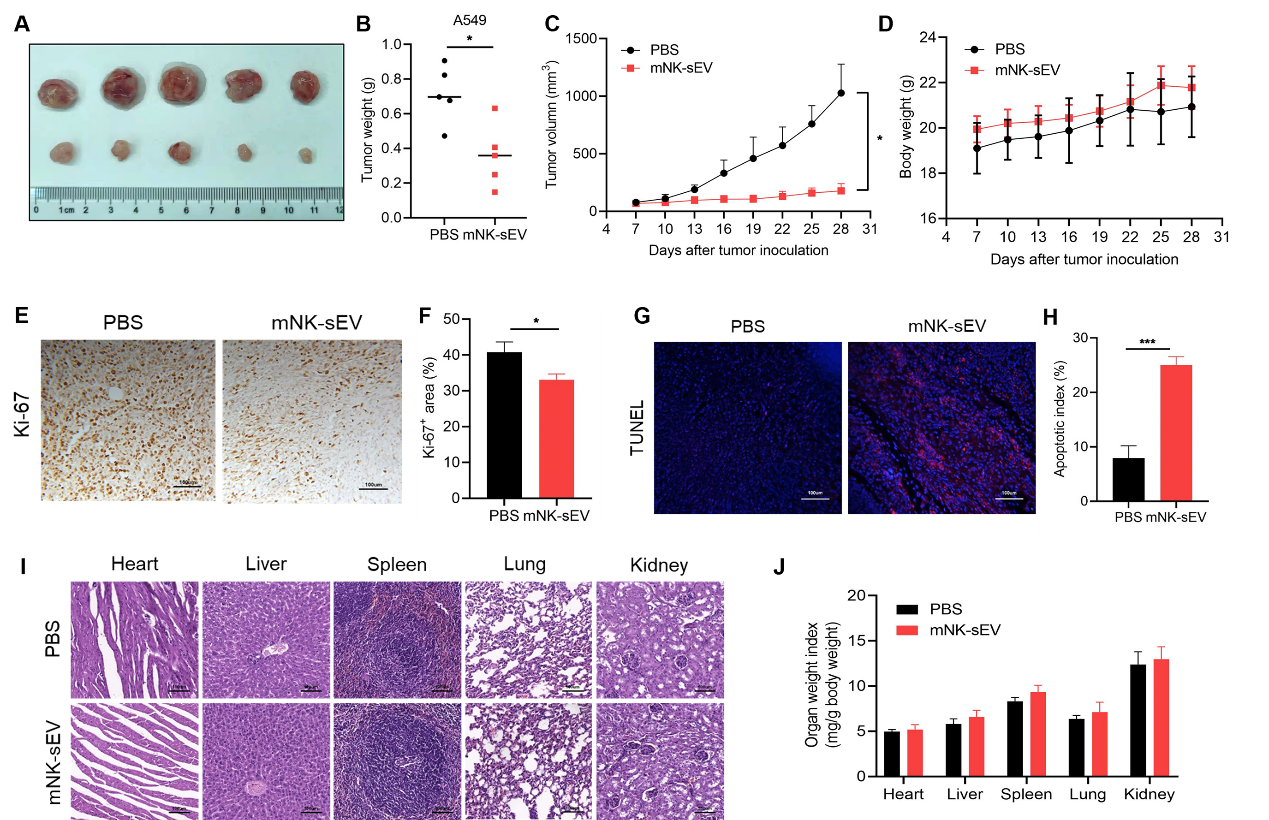
**

**Figure S4.** The antitumor effect of mNK-sEV *in vivo*. (A) Representative images of subcutaneous xenograft tumors established by A549 cells in BALB/c nude mice (n=5 per group) that received PBS or mNK-sEV treatment. (B) Weights of the harvested xenograft tumors at the end of *in vivo* efficacy study. (C) Tumor growth curves under different treatments. (D) Body weights of mice during *in vivo* efficacy study. Body weights of mice were measured every three days. Data are shown as mean ± SD. (E and F) Representative image of Ki-67 staining of tumors from mice (scale bar=100 μm). (G and H) TUNEL staining of tumors from mice (scale bar=100 μm). (I) Representative histological images of main organs (scale bar=100 μm). (J) Major organ weight indexes at the end of the *in vivo* efficacy study. Kidneys, livers, brains, lungs, and hearts from mice were collected and weighted, and organ weight indexes were calculated as organ weight (mg) per gram (g) of mouse body weight. **p*<0.05, ***p*<0.01, ****p*<0.001.

**
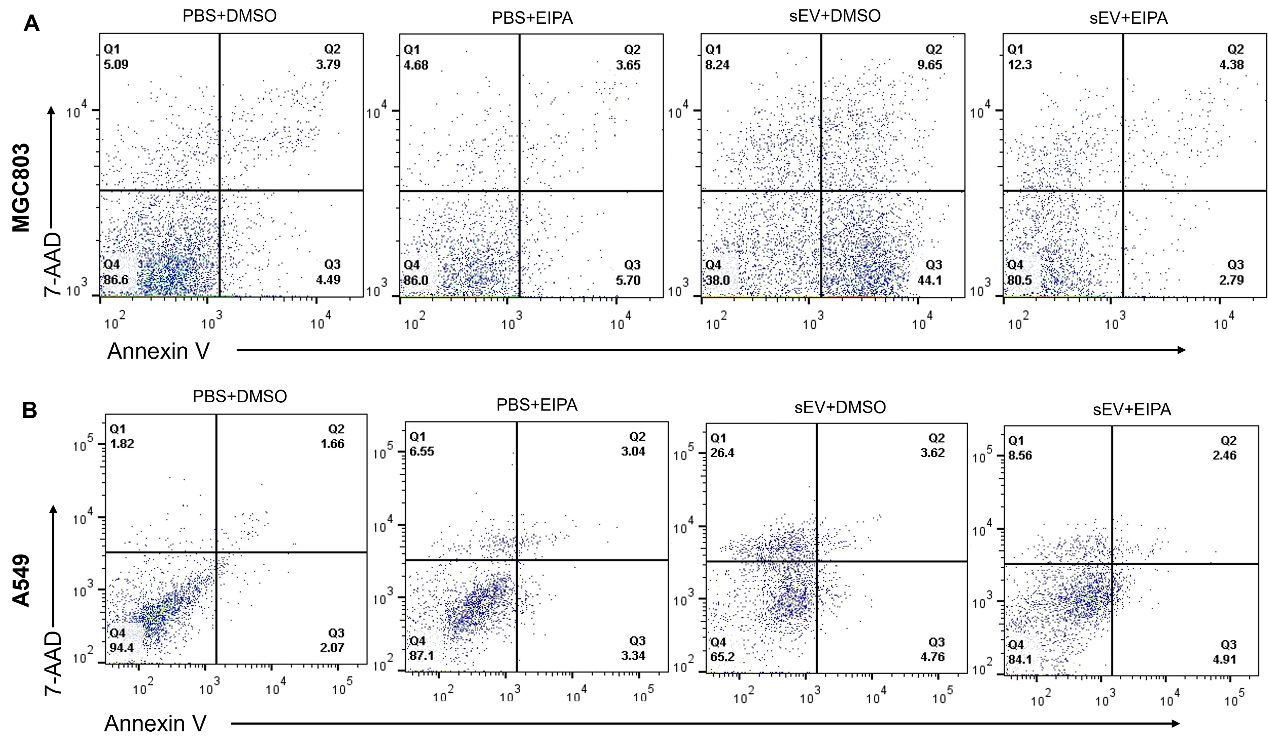
**

**Figure S5.** Representative flow cytometry dot plot for cell apoptosis in (A) MGC803 and (B) A549 cells pre-incubated with DMSO or 100 μM EIPA for 30 min, followed by incubation with PBS or 100 μg/ml mNK-sEV for 24 h.

**
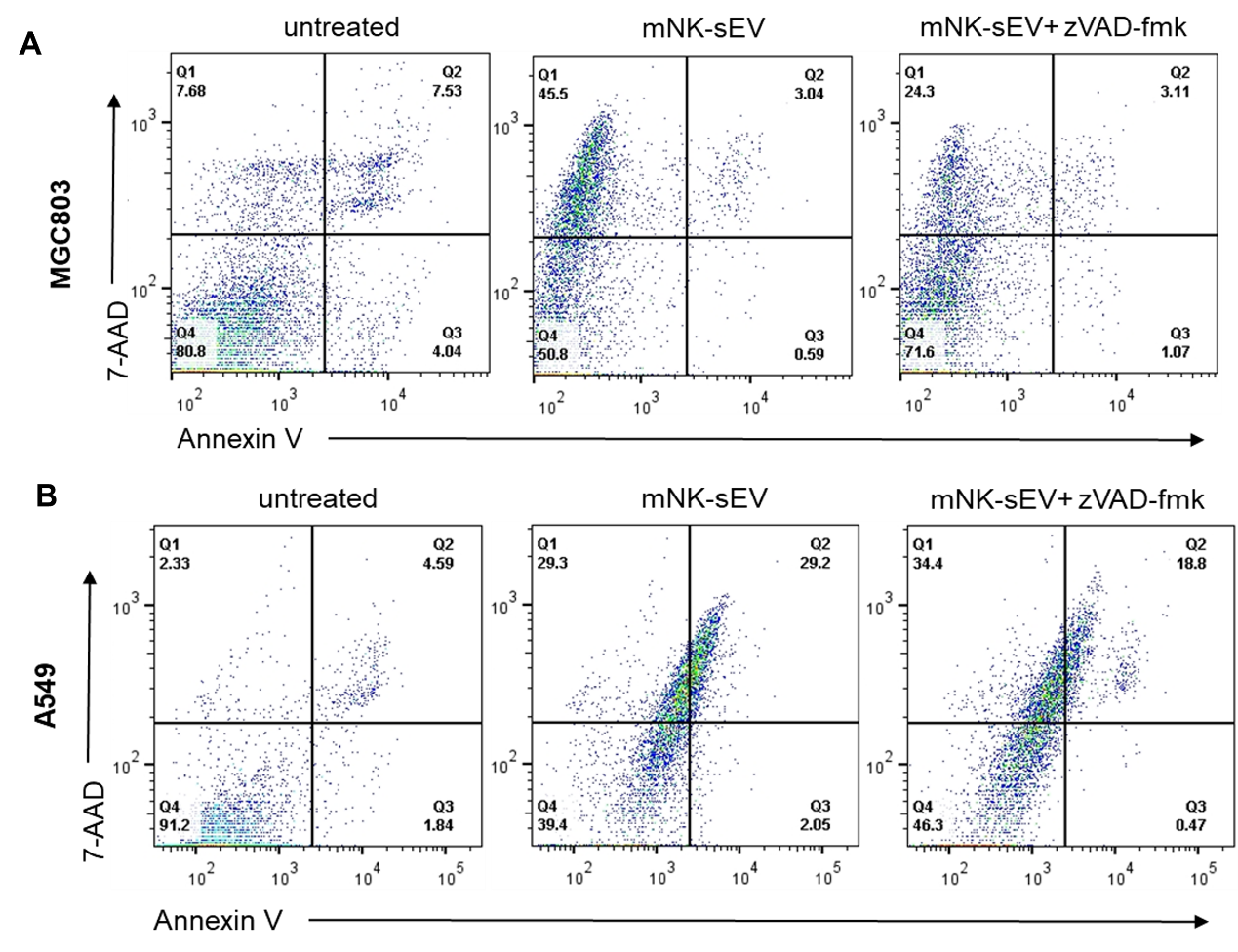
**

**Figure S6.** Representative flow cytometry dot pot for cell apoptosis in (A) MGC803 and (B) A549 cells incubated with 100 μM zVAD-fmk for 30 min, followed by incubation with 100 μg/ml mNK-sEV for 24 h.

**
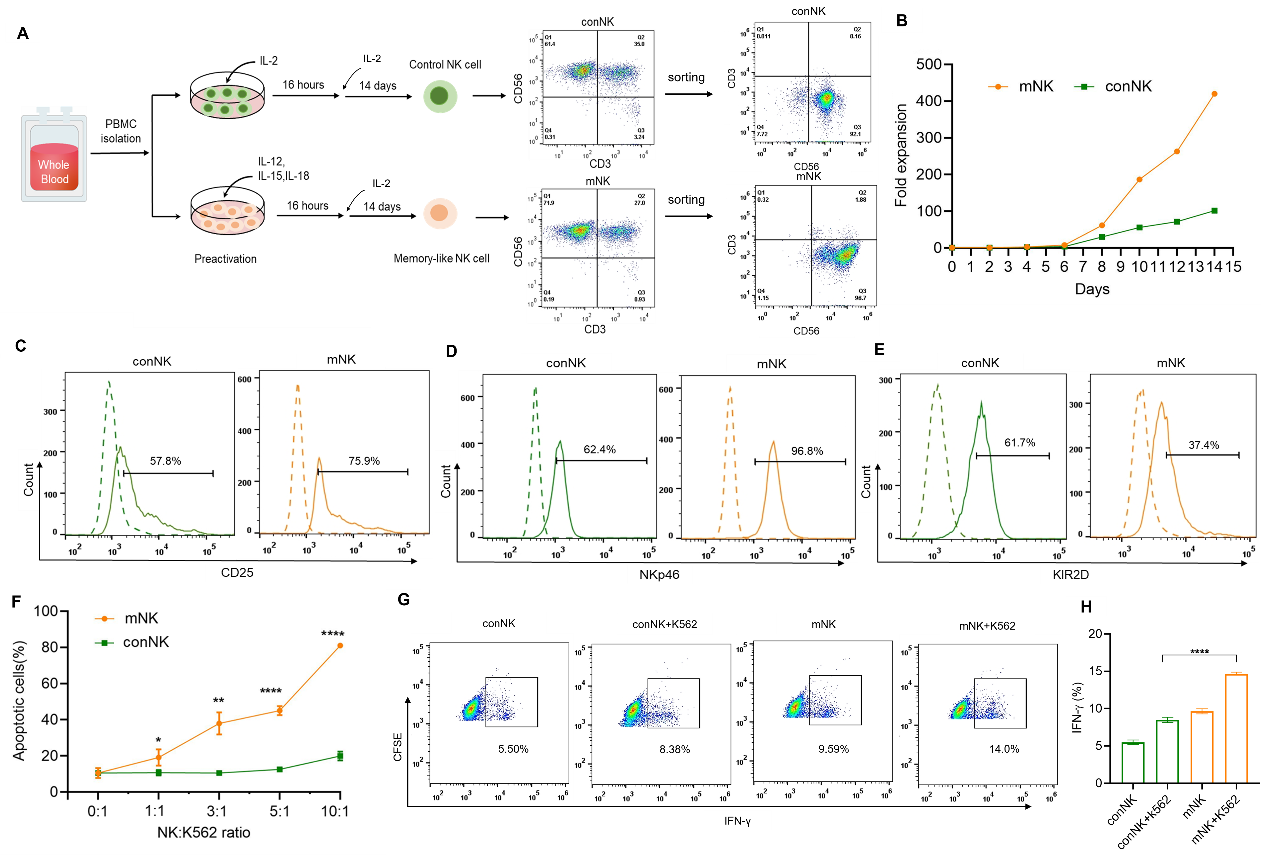
**

**Figure S7.** Characterization of human IL-2 stimulated NK (conNK) cells and IL-12/IL-15/IL-18 stimulated mNK cells. (A) Flow charts for conNK cell and mNK cell culture. (B) Cell growth curve for proliferation of conNK cells and mNK cells. (C, D and E) Representative chart for the expression of CD25, NKp46 and KIR2D in conNK and mNK cells. (F) Representative chart for the percentage of apoptotic K562 cells at various ratios of effector over target by flow cytometry analysis. (G and H) Flow cytometry analysis of IFN-γ secretion in conNK cells and mNK cells when co-cultured with K562 cells, respectively (n=3). **p*<0.05, ***p*<0.01, *****p*<0.0001.

**
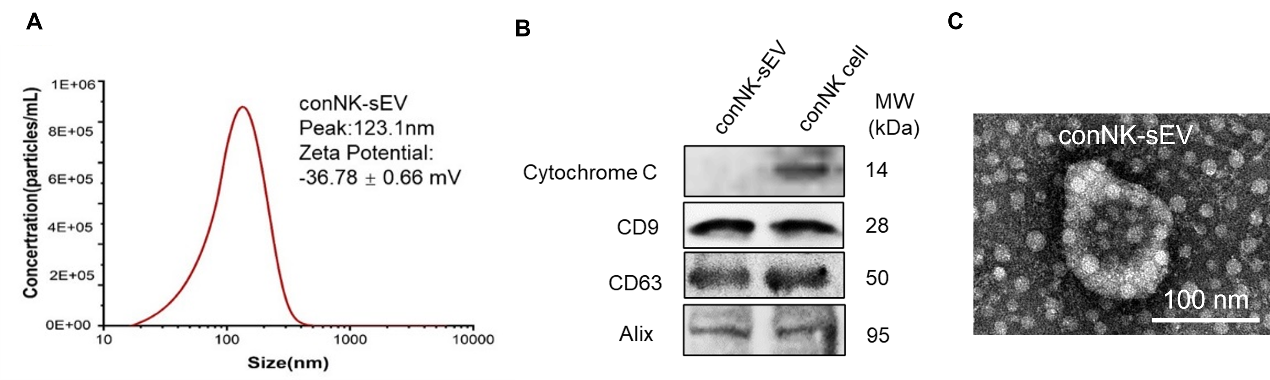
**

**Figure S8.** Characterization of sEV released by IL-2 stimulated conNK cells. (A) The size distribution and surface charge of conNK-sEV were analyzed by NTA. (B) Western blot analysis of CD9, CD63, Alix, and cytochrome c expression in conNK cell lysates and conNK-sEV. (C) conNK-sEV visualized by TEM.

**
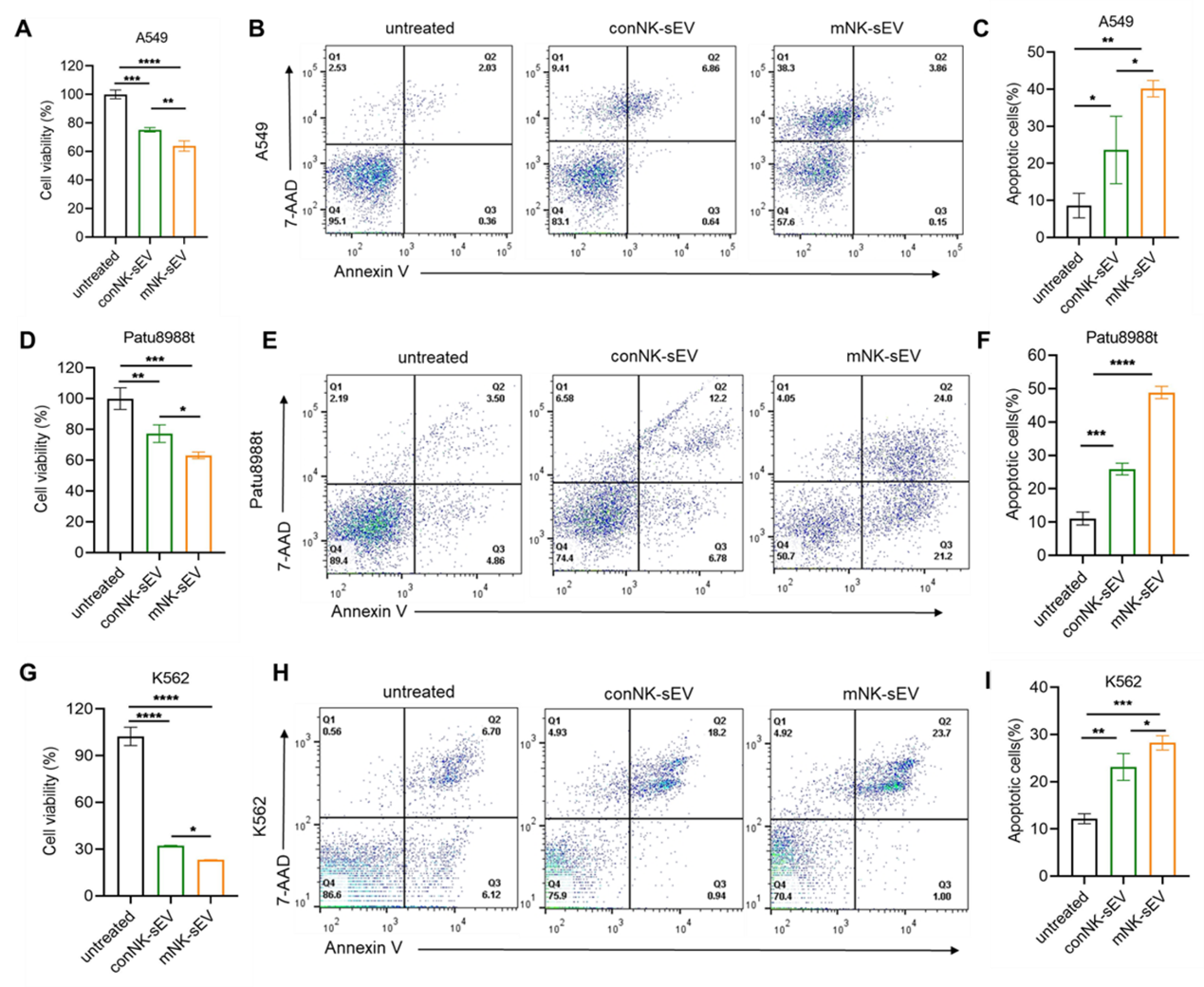
**

**Figure S9.** Cytotoxicity of mNK-sEV was higher than that of conNK-sEV on a variety of tumor cell lines. (A, D, and G) CCK-8 assays for the cytotoxicity of conNK-sEV and mNK-sEV (100 μg/ml) on (A) A549, (D) Patu8988t, and (G) K562 cells at 24 h incubation (n=3). (B, E, and H) Flow cytometry analysis of cell apoptosis in (B)A549, (E) Patu8988t and (H) K562 cells treated with conNK-sEV and mNK-sEV (100 μg/ml) for 24 h. 7-AAD, 7-amino-actinomycin D. (C, F, and I) The percentage of apoptosis in (C) MGC803, (F) Patu8988t and (J) K562 cells (n=3). **p*<0.05, ***p*<0.01, ****p*<0.001, *****p*<0.0001.


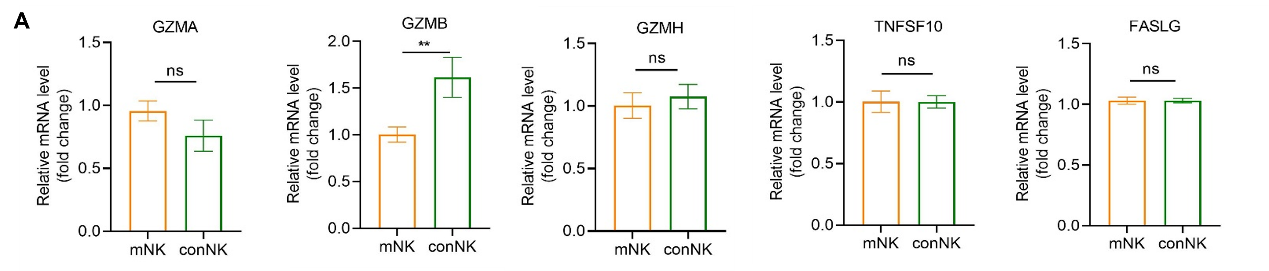


**Figure S10.** qRT-PCR assay for detection of cytotoxicity-related gene expression in conNK cells and mNK cells (n=3). GZMA, granzyme A; GZMB, granzyme B; GZMH, granzyme H; TNFSF10, TRAIL, TNF related apoptosis inducing ligand; FASLG, Fas ligand.
